# Supplementary material for: Trapping of Ag+ into a Perfect Six-Coordinated Environment: Structural Analysis, Quantum Chemical Calculations and Electrochemistry
Source: Molecules. 2022 Oct 17;27(20):6961. doi: 10.3390/molecules27206961 (PMC9607289; doi:10.3390/molecules27206961)
Supplement: Supplementary file 1 [file molecules-27-06961-s001.zip › molecules-1968088-supplementary.pdf]

Trapping of Ag^+ into a perfect six-coordinated environment: structural analysis, quantum chemical calculations and electrochemistry

Veronika I. Komlyagina [1,2], Nikolay F. Romashev [1], Vasily V. Kokovkin [1], Artem L. Gushchin [1,*], Enrico Benassi [2,*], Maxim N. Sokolov [1] and Pavel A. Abramov [1,3,*]

Supplementary Materials

Table of contents

Table S1. Experimental details....2

Table S2. Selected geometric parameters (Å) 3

Figure S1. X-ray powder diffraction patterns comparison....4

Table S3. Shape results 4

Table S4. NBO analysis. Ag-involving interactions. 5

Table S5. Occupancy and composition of Ag NBOs. 6

Table S6. Topological properties computed at the atomic (A), bond (B), ring (R) and cage critical points (C), viz. total electron density (ρtot), Lagrangian kinetic energy (G), Hamiltonian kinetic energy (K), potential energy (V), energy density (H), Laplacian of the total electron density (Δρtot), Source Function (SF), total Electrostatic Potential (EPStot), ellipticity of electron density (ε) and eta index (η) 7

Figure S2. Comparison of CVs of paste electrodes in 0.10 M Bu_4NPF_6 in acetonitrile at a scan rate of 20 mV/s: red curve - complex in paste, blue curve - precursor, green curve - background (blank)....13

Figure S3. Reproducibility of CV curves (2-4 cycles) for **1** in the paste electrode in aqueous solution of 1.0 M $KHCO_3$ at different scan rates (mV/s): blue line – 10, red – 20, green – 50, yellow – 100. 14

Figure S4. The dependences of peak currents on the square root of scan rate for anodic (green circles) and cathodic (blue circles) peak currents. The primary data presented in Figure S3....15

Figure S5. IR spectra of 1 (black curve) and $(Bu_4N)_4[\beta\text{-}Mo_8O_{26}]$ (red curve). 16

Figure S6. TGA data for **1**. 10

Table S1. Experimental details

	**1**
Chemical formula	$C_{104}H_{152}Ag_2Mo_8N_6O_{26}$
M_r	2885.57
Crystal system, space group	Monoclinic, *C*2/*c*
Temperature (K)	150
a, *b*, *c* (Å)	38.5675 (16), 12.6898 (6), 26.2540 (11)
β (°)	116.858 (1)
V (Å^3)	11463.0 (9)
Z	4
Radiation type	Mo *K*a
μ (mm^{-1})	1.25
Crystal size (mm)	0.14 × 0.13 × 0.09
Diffractometer	Bruker D8 Venture diffractometer
Absorption correction	Multi-scan<br>*SADABS* 2016/2: Krause, L., Herbst-Irmer, R., Sheldrick G.M. & Stalke D., J. Appl. Cryst. 48 (2015) 3-10
T_{min}, T_{max}	0.643, 0.746
No. of measured, independent and observed [$I > 2\sigma(I)$] reflections	71574, 18991, 14894
R_{int}	0.042
θ values (°)	θ_{max} = 31.5, θ_{min} = 2.0
$(\sin \theta/\lambda)_{max}$ (Å^{-1})	0.736
Range of *h*, *k*, *l*	$-56 \leq h \leq 56$, $-18 \leq k \leq 14$, $-36 \leq l \leq 38$
$R[F^2 > 2\sigma(F^2)]$, $wR(F^2)$, S	0.036, 0.087, 1.09
No. of reflections, parameters, restraints	18991, 744, 18
H-atom treatment	H-atom parameters constrained
Weighting scheme	$w = 1/[\sigma^2(F_o^2) + (0.03129P)^2 + 1.7353P]$<br>where $P = (F_o^2 + 2F_c^2)/3$
$\Delta\rho_{max}$, $\Delta\rho_{min}$ (e Å^{-3})	0.78, -0.64

Computer programs: *APEX3* (Bruker-AXS, 2016), *SAINT* (Bruker-AXS, 2016), *SHELXT* 2014/5 (Sheldrick, 2014), *SHELXL2017*/1 (Sheldrick, 2017).

Table S2. Selected geometric parameters (Å)

O2—Ag1	2.5693 (17)	O4—Mo3	2.4739 (15)
O6—Ag1	2.5437 (18)	O4—Mo4	2.3195 (15)
O8—Ag1	2.5398 (19)	O5—Mo2	1.6979 (17)
O10—Ag1	2.4948 (17)	O6—Mo2	1.7114 (17)
N1—Ag1	2.461 (2)	O7—Mo2	1.8932 (16)
N2—Ag1	2.386 (2)	O7—Mo3	1.9154 (16)
O1—Mo1	1.7458 (16)	O8—Mo3	1.7127 (18)
O1—Mo3^{i}	2.2973 (17)	O9—Mo3	1.6964 (18)
O2—Mo1	1.7005 (17)	O10—Mo4	1.7074 (17)
O3—Mo1	1.9446 (16)	O11—Mo1	1.9480 (16)
O3—Mo2	2.0061 (16)	O11—Mo2^{i}	2.3092 (16)
O3—Mo4^{i}	2.3020 (16)	O11—Mo4	2.0028 (16)
O4—Mo1	2.1531 (15)	O12—Mo3	1.9204 (17)
O4—Mo1^{i}	2.3343 (15)	O12—Mo4	1.9010 (16)
O4—Mo2	2.3060 (15)	O13—Mo4	1.7000 (17)

Symmetry code(s): (i) -*x*+1, -*y*+1, -*z*+1.

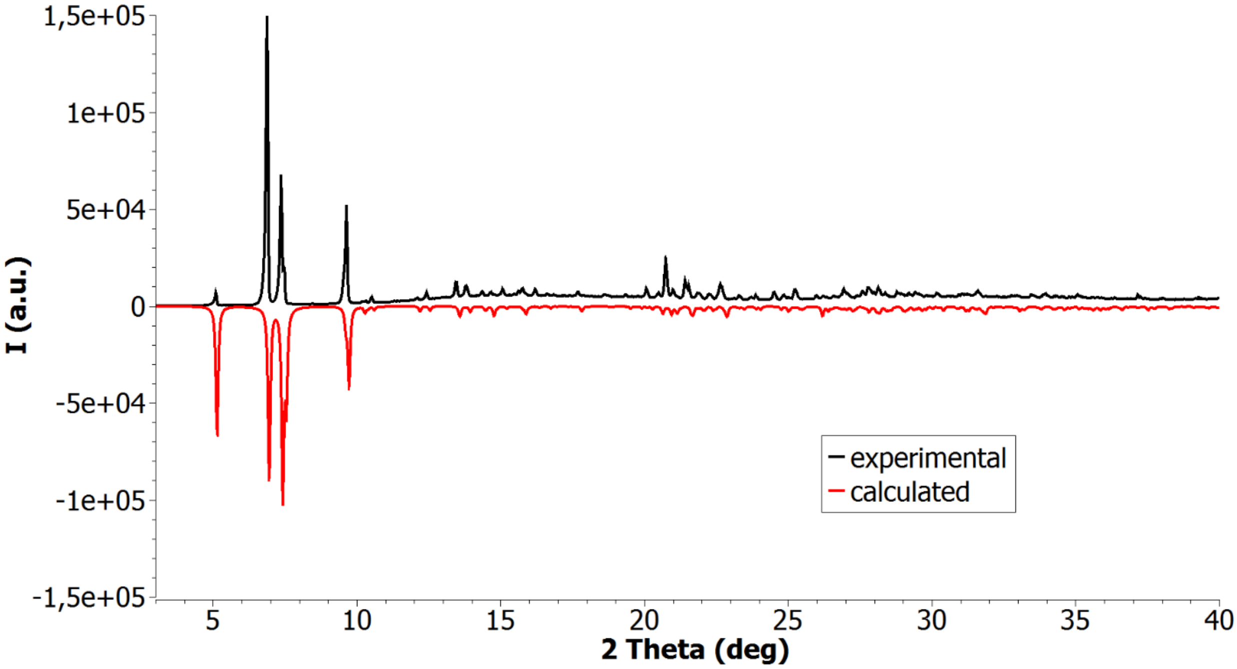

Figure S1. X-ray powder diffraction patterns comparison.

Table S3. Shape results

S H A P E v2.1 Continuous Shape Measures calculation

(c) 2013 Electronic Structure Group, Universitat de Barcelona

Contact: llunell@ub.edu

HP-6	1 D6h	Hexagon
PPY-6	2 C5v	Pentagonal pyramid
OC-6	3 Oh	Octahedron
TPR-6	4 D3h	Trigonal prism
JPPY-6	5 C5v	Johnson pentagonal pyramid J2

Structure [ML6]	HP-6	PPY-6	OC-6	TPR-6	JPPY-6
struct1 ,	30.590,	7.938,	12.027,	7.827,	11.118

Table S4. NBO analysis. Ag-involving interactions.

			Donor								Acceptor				$E^{(2)}$ / (kcal·mol^{-1})
301	CR	(	Ag			)	->	1952	RY*	(	C			)	283.65
432	LP	(	Ag			)	->	1814	RY*	(	C			)	170.37
434	LP	(	Ag			)	->	1579	RY*	(	C			)	63.33
434	LP	(	Ag			)	->	1715	RY*	(	C			)	101.39
434	LP	(	Ag			)	->	1718	RY*	(	C			)	66.67
434	LP	(	Ag			)	->	1751	RY*	(	C			)	78.61
434	LP	(	Ag			)	->	1755	RY*	(	C			)	58.41
434	LP	(	Ag			)	->	1964	RY*	(	C			)	225.68
434	LP	(	Ag			)	->	1970	RY*	(	C			)	190.96
436	LP	(	Ag			)	->	1814	RY*	(	C			)	63.37
303	CR	(	Ag			)	->	2133	BD*	(	C	-	C	)	71.36
421	LP	(	O			)	->	1275	RY*	(	Ag			)	60.77
422	LP	(	O			)	->	1275	RY*	(	Ag			)	90.72
463	LP	(	O			)	->	1275	RY*	(	Ag			)	80.52
458	LP	(	N			)	->	1275	RY*	(	Ag			)	58.55
348	CR	(	C			)	->	1275	RY*	(	Ag			)	83.77
348	CR	(	C			)	->	1276	RY*	(	Ag			)	58.82
348	CR	(	C			)	->	1280	RY*	(	Ag			)	130.10
350	CR	(	C			)	->	1274	RY*	(	Ag			)	55.61
353	CR	(	C			)	->	1276	RY*	(	Ag			)	50.64
353	CR	(	C			)	->	1279	RY*	(	Ag			)	283.48
353	CR	(	C			)	->	1280	RY*	(	Ag			)	199.58
355	CR	(	C			)	->	1275	RY*	(	Ag			)	183.46
355	CR	(	C			)	->	1278	RY*	(	Ag			)	115.75
355	CR	(	C			)	->	1279	RY*	(	Ag			)	538.10
355	CR	(	C			)	->	1280	RY*	(	Ag			)	389.56
359	CR	(	C			)	->	1274	RY*	(	Ag			)	102.35
359	CR	(	C			)	->	1280	RY*	(	Ag			)	70.78
366	CR	(	C			)	->	1274	RY*	(	Ag			)	59.77
366	CR	(	C			)	->	1279	RY*	(	Ag			)	104.30
366	CR	(	C			)	->	1280	RY*	(	Ag			)	128.04
367	CR	(	C			)	->	1275	RY*	(	Ag			)	151.82
368	CR	(	C			)	->	1278	RY*	(	Ag			)	72.95
368	CR	(	C			)	->	1279	RY*	(	Ag			)	149.13
368	CR	(	C			)	->	1280	RY*	(	Ag			)	69.20
311	CR	(	Mo			)	->	1280	RY*	(	Ag			)	275.42
315	CR	(	Mo			)	->	1280	RY*	(	Ag			)	60.83
246	CR	(	Mo			)	->	1280	RY*	(	Ag			)	60.98
25	BD	(	N	-	C	)	->	1275	RY*	(	Ag			)	110.62
136	BD	(	N	-	C	)	->	1275	RY*	(	Ag			)	118.61
138	BD	(	N	-	C	)	->	1279	RY*	(	Ag			)	59.27
28	BD	(	C	-	C	)	->	1275	RY*	(	Ag			)	144.27
34	BD	(	C	-	C	)	->	440	LP*	(	Ag			)	64.94
50	BD	(	C	-	C	)	->	440	LP*	(	Ag			)	115.07
50	BD	(	C	-	C	)	->	1275	RY*	(	Ag			)	320.45
72	BD	(	C	-	C	)	->	1276	RY*	(	Ag			)	51.11
151	BD	(	C	-	C	)	->	1275	RY*	(	Ag			)	64.25
154	BD	(	C	-	C	)	->	1275	RY*	(	Ag			)	216.24
154	BD	(	C	-	C	)	->	1276	RY*	(	Ag			)	52.20
157	BD	(	C	-	C	)	->	1275	RY*	(	Ag			)	54.47
165	BD	(	C	-	C	)	->	1275	RY*	(	Ag			)	300.69
172	BD	(	C	-	C	)	->	439	LP*	(	Ag			)	61.23
172	BD	(	C	-	C	)	->	440	LP*	(	Ag			)	82.90
172	BD	(	C	-	C	)	->	1274	RY*	(	Ag			)	91.68
172	BD	(	C	-	C	)	->	1275	RY*	(	Ag			)	247.82
172	BD	(	C	-	C	)	->	1276	RY*	(	Ag			)	101.12
173	BD	(	C	-	C	)	->	1275	RY*	(	Ag			)	120.52
177	BD	(	C	-	C	)	->	438	LP*	(	Ag			)	63.45
177	BD	(	C	-	C	)	->	440	LP*	(	Ag			)	75.53
177	BD	(	C	-	C	)	->	1275	RY*	(	Ag			)	115.52
178	BD	(	C	-	C	)	->	439	LP*	(	Ag			)	54.96
178	BD	(	C	-	C	)	->	440	LP*	(	Ag			)	74.31
178	BD	(	C	-	C	)	->	1274	RY*	(	Ag			)	57.11
185	BD	(	C	-	C	)	->	1275	RY*	(	Ag			)	116.60
186	BD	(	C	-	C	)	->	439	LP*	(	Ag			)	71.05
186	BD	(	C	-	C	)	->	440	LP*	(	Ag			)	81.45
186	BD	(	C	-	C	)	->	1276	RY*	(	Ag			)	92.96
190	BD	(	C	-	C	)	->	439	LP*	(	Ag			)	66.61
190	BD	(	C	-	C	)	->	440	LP*	(	Ag			)	72.86
191	BD	(	C	-	C	)	->	438	LP*	(	Ag			)	59.52
191	BD	(	C	-	C	)	->	440	LP*	(	Ag			)	81.15
191	BD	(	C	-	C	)	->	1275	RY*	(	Ag			)	413.77
94	BD	(	C	-	H	)	->	440	LP*	(	Ag			)	51.51
94	BD	(	C	-	H	)	->	1275	RY*	(	Ag			)	135.33
174	BD	(	C	-	H	)	->	438	LP*	(	Ag			)	50.01
179	BD	(	C	-	H	)	->	440	LP*	(	Ag			)	762.85
179	BD	(	C	-	H	)	->	1274	RY*	(	Ag			)	89.45
187	BD	(	C	-	H	)	->	438	LP*	(	Ag			)	50.32
189	BD	(	C	-	H	)	->	440	LP*	(	Ag			)	69.08
192	BD	(	C	-	H	)	->	440	LP*	(	Ag			)	327.47
199	BD	(	C	-	H	)	->	439	LP*	(	Ag			)	70.35
199	BD	(	C	-	H	)	->	440	LP*	(	Ag			)	665.84
199	BD	(	C	-	H	)	->	1274	RY*	(	Ag			)	51.17
200	BD	(	C	-	H	)	->	439	LP*	(	Ag			)	124.55
200	BD	(	C	-	H	)	->	440	LP*	(	Ag			)	243.32
200	BD	(	C	-	H	)	->	1274	RY*	(	Ag			)	86.05
200	BD	(	C	-	H	)	->	1275	RY*	(	Ag			)	1321.85
201	BD	(	C	-	H	)	->	439	LP*	(	Ag			)	82.96
201	BD	(	C	-	H	)	->	440	LP*	(	Ag			)	344.47
205	BD	(	C	-	H	)	->	438	LP*	(	Ag			)	74.22
205	BD	(	C	-	H	)	->	440	LP*	(	Ag			)	145.72
206	BD	(	C	-	H	)	->	440	LP*	(	Ag			)	73.41

207	BD	(	C	-	H	)	->	438	LP*	(	Ag		)	57.13
207	BD	(	C	-	H	)	->	440	LP*	(	Ag		)	154.75
208	BD	(	C	-	H	)	->	440	LP*	(	Ag		)	211.17
209	BD	(	C	-	H	)	->	439	LP*	(	Ag		)	59.85
209	BD	(	C	-	H	)	->	440	LP*	(	Ag		)	135.76
210	BD	(	C	-	H	)	->	439	LP*	(	Ag		)	51.37
210	BD	(	C	-	H	)	->	440	LP*	(	Ag		)	173.22
218	BD	(	C	-	H	)	->	439	LP*	(	Ag		)	87.38
218	BD	(	C	-	H	)	->	440	LP*	(	Ag		)	315.74
219	BD	(	C	-	H	)	->	439	LP*	(	Ag		)	144.68
219	BD	(	C	-	H	)	->	440	LP*	(	Ag		)	243.78
219	BD	(	C	-	H	)	->	1274	RY*	(	Ag		)	57.51
220	BD	(	C	-	H	)	->	439	LP*	(	Ag		)	74.15
220	BD	(	C	-	H	)	->	440	LP*	(	Ag		)	629.71
221	BD	(	C	-	H	)	->	440	LP*	(	Ag		)	212.53
222	BD	(	C	-	H	)	->	439	LP*	(	Ag		)	63.63
222	BD	(	C	-	H	)	->	440	LP*	(	Ag		)	170.00
224	BD	(	C	-	H	)	->	438	LP*	(	Ag		)	81.58
224	BD	(	C	-	H	)	->	440	LP*	(	Ag		)	124.06
225	BD	(	C	-	H	)	->	438	LP*	(	Ag		)	50.78
225	BD	(	C	-	H	)	->	440	LP*	(	Ag		)	197.43
226	BD	(	C	-	H	)	->	440	LP*	(	Ag		)	83.67

Table S5. Occupancy and composition of Ag NBOs.

					occ.	s%	p%	d%
301	CR	(	Ag	)	1.99822	0.00	100.00	0.00
303	CR	(	Ag	)	1.99913	0.00	100.00	0.00
432	LP	(	Ag	)	1.99601	0.00	0.00	100.00
434	LP	(	Ag	)	1.99323	0.05	0.04	99.92
436	LP	(	Ag	)	1.99039	0.00	0.01	99.99
437	LP*	(	Ag	)	0.11626	64.99	34.99	0.02
438	LP*	(	Ag	)	0.09007	32.91	66.90	0.18
439	LP*	(	Ag	)	0.07325	0.00	99.97	0.03
440	LP*	(	Ag	)	0.05996	1.98	98.00	0.03
1274	RY*	(	Ag	)	0.00118	0.49	25.08	74.43
1275	RY*	(	Ag	)	0.00096	2.56	75.86	21.58
1276	RY*	(	Ag	)	0.00069	4.60	30.98	64.42
1278	RY*	(	Ag	)	0.00041	60.42	7.19	32.39
1279	RY*	(	Ag	)	0.00032	22.35	27.35	50.30
1280	RY*	(	Ag	)	0.00010	0.02	0.78	99.20

Table S6 Topological properties computed at the atomic (A), bond (B), ring (R) and cage critical points (C), *viz.* total electron density (ρ_{tot}), Lagrangian kinetic energy (G), Hamiltonian kinetic energy (K), potential energy (V), energy density (H), Laplacian of the total electron density ($\Delta\rho_{tot}$), Source Function (SF), total Electrostatic Potential (EPS_{tot}), ellipticity of electron density (ε) and eta index (η).

#	type	ρ_{tot}(**r**)	G(**r**)	K(**r**)	V(**r**)	H(**r**)	$\nabla^2\rho_{tot}$(**r**)	SF(**r**)	ESP_{tot}(**r**)	ε(**r**)	η(**r**)
1	A	3.716E-01	5.269E-03	1.956E-01	-1.961E+10	-1.956E+00	-7.804E+00	3.754E-02	1.108E+01	0.002584	-1.229003
2	A	3.726E-01	5.547E-03	1.960E-01	-1.966E+10	-1.960E+00	-7.819E+00	3.906E-02	1.106E+01	0.002628	-1.229392
3	A	3.700E-01	5.816E-03	1.954E-01	-1.960E+10	-1.954E+00	-7.792E+00	3.684E-02	1.157E+01	0.000521	-1.212999
4	A	1.181E+02	5.186E+00	1.092E-01	-1.092E+65	-1.092E+05	-4.368E+05	2.274E+03	1.725E+07	0.000006	-1.000008
5	A	1.181E+02	5.207E+00	1.092E-01	-1.092E+65	-1.092E+05	-4.369E+05	2.330E+03	1.600E+07	0.000006	-1.000007
6	A	3.716E-01	5.269E-03	1.956E-01	-1.961E+10	-1.956E+00	-7.804E+00	3.753E-02	1.108E+01	0.002584	-1.228999
7	A	3.693E-01	5.704E-03	1.950E-01	-1.956E+10	-1.950E+00	-7.777E+00	3.452E-02	1.160E+01	0.000616	-1.212322
8	A	1.181E+02	5.220E+00	1.092E-01	-1.092E+65	-1.092E+05	-4.366E+05	2.109E+03	2.061E+07	0.000001	-1.000001
9	A	3.718E-01	5.463E-03	1.952E-01	-1.958E+10	-1.952E+00	-7.788E+00	4.104E-02	1.088E+01	0.002529	-1.233744
10	A	3.718E-01	5.462E-03	1.952E-01	-1.958E+10	-1.952E+00	-7.788E+00	4.103E-02	1.088E+01	0.002529	-1.233744
11	A	1.182E+02	5.208E+00	1.092E-01	-1.092E+65	-1.092E+05	-4.369E+05	2.411E+03	1.143E+07	0.000007	-1.000007
12	A	3.692E-01	5.689E-03	1.949E-01	-1.954E+10	-1.949E+00	-7.772E+00	3.651E-02	1.155E+01	0.000582	-1.213512
13	A	3.683E-01	5.674E-03	1.944E-01	-1.950E+10	-1.944E+00	-7.755E+00	5.085E-02	1.143E+01	0.000861	-1.215215
14	A	1.181E+02	5.186E+00	1.092E-01	-1.092E+65	-1.092E+05	-4.368E+05	2.273E+03	1.726E+07	0.000006	-1.000008
15	A	3.709E-01	5.174E-03	1.947E-01	-1.953E+10	-1.947E+00	-7.769E+00	2.776E-02	1.091E+01	0.002608	-1.234633
16	A	1.181E+02	5.191E+00	1.092E-01	-1.092E+65	-1.092E+05	-4.369E+05	2.537E+03	8.452E+06	0.000005	-1.000007
17	A	3.660E-01	5.319E-03	1.905E-01	-1.911E+10	-1.905E+00	-7.600E+00	3.387E-02	1.023E+01	0.002080	-1.252835
18	A	1.182E+02	5.208E+00	1.092E-01	-1.092E+65	-1.092E+05	-4.369E+05	2.410E+03	1.143E+07	0.000007	-1.000007
19	A	3.711E-01	5.881E-03	1.968E-01	-1.974E+10	-1.968E+00	-7.848E+00	4.305E-02	1.194E+01	0.000760	-1.205035
20	A	1.180E+02	5.159E+00	1.091E-01	-1.091E+65	-1.091E+05	-4.364E+05	2.508E+03	4.108E+06	0.000002	-1.000003
21	A	3.726E-01	5.547E-03	1.960E-01	-1.966E+10	-1.960E+00	-7.819E+00	3.905E-02	1.106E+01	0.002629	-1.229403
22	A	2.961E+02	6.799E+01	6.174E-01	-6.175E+65	-6.174E+05	-2.469E+06	2.402E+04	1.556E+06	0.000010	-1.000010
23	A	1.181E+02	5.240E+00	1.092E-01	-1.092E+65	-1.092E+05	-4.368E+05	2.940E+03	2.967E+06	0.000003	-1.000005
24	A	2.961E+02	6.799E+01	6.174E-01	-6.175E+65	-6.174E+05	-2.469E+06	2.402E+04	1.556E+06	0.000010	-1.000010
25	A	1.181E+02	5.207E+00	1.092E-01	-1.092E+65	-1.092E+05	-4.369E+05	2.330E+03	1.600E+07	0.000006	-1.000007
26	A	1.181E+02	5.164E+00	1.092E-01	-1.092E+65	-1.092E+05	-4.367E+05	1.659E+03	1.138E+07	0.000008	-1.000008
27	A	1.182E+02	5.180E+00	1.092E-01	-1.092E+65	-1.092E+05	-4.369E+05	2.648E+03	5.160E+06	0.000006	-1.000009
28	A	3.660E-01	5.628E-03	1.918E-01	-1.924E+10	-1.918E+00	-7.650E+00	5.384E-02	1.076E+01	0.000686	-1.230327
29	A	3.690E-01	5.663E-03	1.951E-01	-1.956E+10	-1.951E+00	-7.780E+00	4.995E-02	1.158E+01	0.000886	-1.212347
30	A	1.182E+02	5.190E+00	1.092E-01	-1.092E+65	-1.092E+05	-4.369E+05	1.897E+03	4.148E+06	0.000008	-1.000010
31	A	1.181E+02	5.183E+00	1.092E-01	-1.092E+65	-1.092E+05	-4.368E+05	2.710E+03	1.234E+06	0.000005	-1.000009
32	A	3.711E-01	5.881E-03	1.968E-01	-1.974E+10	-1.968E+00	-7.848E+00	4.305E-02	1.194E+01	0.000760	-1.205034
33	A	1.181E+02	5.246E+00	1.092E-01	-1.092E+65	-1.092E+05	-4.367E+05	2.728E+03	4.037E+06	0.000002	-1.000004
34	A	3.781E-01	6.172E-03	1.997E-01	-2.003E+10	-1.997E+00	-7.962E+00	4.541E-02	1.154E+01	0.000400	-1.216387
35	A	3.642E-01	5.612E-03	1.905E-01	-1.911E+10	-1.905E+00	-7.599E+00	6.077E-02	1.054E+01	0.000652	-1.236361
36	A	3.691E-01	5.746E-03	1.952E-01	-1.958E+10	-1.952E+00	-7.786E+00	3.668E-02	1.171E+01	0.000583	-1.210026
37	A	3.691E-01	5.745E-03	1.952E-01	-1.958E+10	-1.952E+00	-7.786E+00	3.668E-02	1.171E+01	0.000583	-1.210026
38	A	3.701E-01	5.297E-03	1.946E-01	-1.951E+10	-1.946E+00	-7.763E+00	2.576E-02	1.102E+01	0.002351	-1.231681
39	A	1.182E+02	5.180E+00	1.092E-01	-1.092E+65	-1.092E+05	-4.369E+05	2.648E+03	5.160E+06	0.000006	-1.000009
40	A	3.679E-01	5.642E-03	1.937E-01	-1.943E+10	-1.937E+00	-7.726E+00	4.801E-02	1.123E+01	0.000791	-1.219407
41	A	3.659E-01	5.628E-03	1.918E-01	-1.924E+10	-1.918E+00	-7.650E+00	5.384E-02	1.076E+01	0.000686	-1.230341
42	A	1.181E+02	5.174E+00	1.092E-01	-1.092E+65	-1.092E+05	-4.368E+05	1.579E+03	1.177E+07	0.000007	-1.000008
43	A	1.181E+02	5.246E+00	1.092E-01	-1.092E+65	-1.092E+05	-4.367E+05	2.728E+03	4.036E+06	0.000002	-1.000004
44	A	3.700E-01	5.816E-03	1.954E-01	-1.960E+10	-1.954E+00	-7.792E+00	3.684E-02	1.157E+01	0.000521	-1.213009
45	A	1.180E+02	5.193E+00	1.091E-01	-1.091E+65	-1.091E+05	-4.365E+05	2.705E+03	2.257E+06	0.000002	-1.000006
46	A	2.959E+02	6.856E+01	6.171E-01	-6.172E+65	-6.171E+05	-2.468E+06	2.660E+04	1.713E+06	0.000007	-1.000011
47	A	1.180E+02	5.193E+00	1.091E-01	-1.091E+65	-1.091E+05	-4.365E+05	2.705E+03	2.258E+06	0.000002	-1.000006
48	A	1.181E+02	5.191E+00	1.092E-01	-1.092E+65	-1.092E+05	-4.369E+05	2.536E+03	8.447E+06	0.000005	-1.000007
49	C	2.314E-05	2.352E-01	-2.045E-01	-2.148E+00	2.045E-02	1.023E+00	-1.477E-02	2.194E+04	-0.013707	0.972549
50	C	2.314E-05	2.352E-01	-2.045E-01	-2.148E+00	2.045E-02	1.023E+00	-1.477E-02	2.193E+04	-0.013692	0.972549
51	A	1.182E+02	5.263E+00	1.092E-01	-1.092E+65	-1.092E+05	-4.370E+05	2.082E+03	6.488E+06	0.000006	-1.000008
52	A	2.955E+02	6.962E+01	6.163E-01	-6.164E+65	-6.163E+05	-2.465E+06	2.888E+04	1.823E+06	0.000010	-1.000015
53	A	1.181E+02	5.209E+00	1.092E-01	-1.092E+65	-1.092E+05	-4.368E+05	2.262E+03	1.679E+07	0.000001	-1.000002
54	A	2.955E+02	6.987E+01	6.162E-01	-6.162E+65	-6.162E+05	-2.464E+06	4.544E+04	2.016E+06	0.000004	-1.000013
55	A	1.181E+02	5.209E+00	1.092E-01	-1.092E+65	-1.092E+05	-4.368E+05	2.262E+03	1.677E+07	0.000001	-1.000002
56	A	1.943E+02	2.574E+01	3.034E-01	-3.035E+65	-3.034E+05	-1.214E+06	7.968E+03	7.270E+05	0.000006	-1.000032
57	A	3.660E-01	5.319E-03	1.905E-01	-1.911E+10	-1.905E+00	-7.600E+00	3.386E-02	1.023E+01	0.002080	-1.252837
58	A	3.713E-01	5.843E-03	1.962E-01	-1.968E+10	-1.962E+00	-7.826E+00	3.926E-02	1.159E+01	0.000464	-1.212212
59	A	3.683E-01	5.674E-03	1.944E-01	-1.950E+10	-1.944E+00	-7.754E+00	5.086E-02	1.143E+01	0.000862	-1.215240
60	A	1.181E+02	5.183E+00	1.092E-01	-1.092E+65	-1.092E+05	-4.368E+05	2.710E+03	1.235E+06	0.000005	-1.000009
61	A	3.693E-01	5.704E-03	1.950E-01	-1.956E+10	-1.950E+00	-7.777E+00	3.451E-02	1.160E+01	0.000616	-1.212321
62	A	2.959E+02	6.856E+01	6.171E-01	-6.172E+65	-6.171E+05	-2.468E+06	2.660E+04	1.713E+06	0.000007	-1.000011
63	A	3.709E-01	5.174E-03	1.947E-01	-1.953E+10	-1.947E+00	-7.769E+00	2.775E-02	1.091E+01	0.002608	-1.234632
64	A	3.679E-01	5.642E-03	1.937E-01	-1.943E+10	-1.937E+00	-7.726E+00	4.800E-02	1.123E+01	0.000791	-1.219397
65	A	3.683E-01	5.657E-03	1.942E-01	-1.948E+10	-1.942E+00	-7.746E+00	3.962E-02	1.144E+01	0.000591	-1.215553
66	A	1.181E+02	5.220E+00	1.092E-01	-1.092E+65	-1.092E+05	-4.366E+05	2.108E+03	2.055E+07	0.000001	-1.000001
67	A	1.182E+02	5.161E+00	1.093E-01	-1.093E+65	-1.093E+05	-4.371E+05	2.493E+03	1.065E+07	0.000007	-1.000009
68	A	3.691E-01	6.393E-03	1.930E-01	-1.936E+10	-1.930E+00	-7.694E+00	5.376E-02	1.061E+01	0.000274	-1.237793
69	A	3.691E-01	6.392E-03	1.930E-01	-1.936E+10	-1.930E+00	-7.694E+00	5.376E-02	1.061E+01	0.000275	-1.237788
70	A	2.955E+02	6.987E+01	6.162E-01	-6.162E+65	-6.162E+05	-2.464E+06	4.544E+04	2.016E+06	0.000004	-1.000013
71	A	3.713E-01	5.843E-03	1.962E-01	-1.968E+10	-1.962E+00	-7.826E+00	3.926E-02	1.159E+01	0.000464	-1.212208

72	A	1.181E+02	5.242E+00	1.092E-01	-1.092E+65	-1.092E+05	-4.366E+05	1.679E+03	1.379E+08	0.000008	-1.000008
73	A	1.180E+02	5.159E+00	1.091E-01	-1.091E+65	-1.091E+05	-4.364E+05	2.507E+03	4.111E+06	0.000002	-1.000003
74	A	3.683E-01	5.657E-03	1.942E-01	-1.948E+10	-1.942E+00	-7.746E+00	3.962E-02	1.144E+01	0.000591	-1.215539
75	A	2.955E+02	6.962E+01	6.163E-01	-6.164E+65	-6.163E+05	-2.465E+06	2.888E+04	1.823E+06	0.000010	-1.000015
76	A	1.182E+02	5.280E+00	1.093E-01	-1.093E+65	-1.093E+05	-4.370E+05	1.929E+03	3.124E+07	0.000010	-1.000010
77	A	1.181E+02	5.240E+00	1.092E-01	-1.092E+65	-1.092E+05	-4.368E+05	2.939E+03	2.968E+06	0.000003	-1.000005
78	A	1.182E+02	5.190E+00	1.092E-01	-1.092E+65	-1.092E+05	-4.369E+05	1.897E+03	4.148E+06	0.000008	-1.000010
79	A	2.960E+02	6.806E+01	6.173E-01	-6.174E+65	-6.173E+05	-2.469E+06	1.964E+04	1.544E+06	0.000002	-1.000006
80	A	1.181E+02	5.164E+00	1.092E-01	-1.092E+65	-1.092E+05	-4.367E+05	1.659E+03	1.138E+07	0.000008	-1.000008
81	A	3.692E-01	5.689E-03	1.949E-01	-1.954E+10	-1.949E+00	-7.772E+00	3.650E-02	1.155E+01	0.000582	-1.213516
82	A	3.642E-01	5.613E-03	1.906E-01	-1.911E+10	-1.906E+00	-7.600E+00	6.075E-02	1.054E+01	0.000652	-1.236334
83	A	2.959E+02	6.841E+01	6.172E-01	-6.172E+65	-6.172E+05	-2.468E+06	3.161E+04	1.766E+06	0.000005	-1.000008
84	A	2.958E+02	6.888E+01	6.169E-01	-6.170E+65	-6.169E+05	-2.467E+06	3.462E+04	2.082E+06	0.000007	-1.000010
85	A	1.943E+02	2.574E+01	3.034E-01	-3.035E+65	-3.034E+05	-1.214E+06	7.967E+03	7.269E+05	0.000006	-1.000032
86	C	2.569E-05	2.365E-01	-2.183E-01	-2.146E+00	2.183E-02	1.033E+00	-1.160E-02	2.064E+04	-0.013878	0.974967
87	C	2.210E-05	2.325E-01	-2.011E-01	-2.124E+00	2.011E-02	1.011E+00	-2.392E-02	5.032E+04	-0.001977	0.974640
88	C	1.592E-04	7.518E-01	-1.461E-01	-6.056E-01	1.461E-01	3.592E+00	-3.580E-02	1.284E+05	-0.012590	0.986042
89	A	3.781E-01	6.171E-03	1.997E-01	-2.003E+10	-1.997E+00	-7.962E+00	4.538E-02	1.154E+01	0.000400	-1.216363
90	A	3.701E-01	5.297E-03	1.946E-01	-1.951E+10	-1.946E+00	-7.763E+00	2.576E-02	1.102E+01	0.002351	-1.231681
91	A	3.690E-01	5.663E-03	1.951E-01	-1.956E+10	-1.951E+00	-7.780E+00	4.994E-02	1.158E+01	0.000887	-1.212348
92	A	1.181E+02	5.174E+00	1.092E-01	-1.092E+65	-1.092E+05	-4.368E+05	1.579E+03	1.177E+07	0.000007	-1.000008
93	A	2.959E+02	6.863E+01	6.170E-01	-6.171E+65	-6.170E+05	-2.468E+06	2.277E+04	1.773E+06	0.000006	-1.000008
94	A	1.182E+02	5.160E+00	1.093E-01	-1.093E+65	-1.093E+05	-4.371E+05	2.493E+03	1.064E+07	0.000007	-1.000009
95	A	1.182E+02	5.263E+00	1.092E-01	-1.092E+65	-1.092E+05	-4.370E+05	2.082E+03	6.487E+06	0.000006	-1.000008
96	A	2.953E+02	7.003E+01	6.159E-01	-6.160E+65	-6.159E+05	-2.463E+06	7.609E+04	4.897E+06	0.000003	-1.000008
97	A	2.953E+02	7.003E+01	6.159E-01	-6.160E+65	-6.159E+05	-2.463E+06	7.608E+04	4.898E+06	0.000003	-1.000008
98	A	1.182E+02	5.263E+00	1.092E-01	-1.092E+65	-1.092E+05	-4.370E+05	2.082E+03	6.487E+06	0.000006	-1.000008
99	A	1.182E+02	5.160E+00	1.093E-01	-1.093E+65	-1.093E+05	-4.371E+05	2.493E+03	1.065E+07	0.000007	-1.000009
100	A	2.959E+02	6.863E+01	6.170E-01	-6.171E+65	-6.170E+05	-2.468E+06	2.277E+04	1.773E+06	0.000006	-1.000008
101	A	1.181E+02	5.174E+00	1.092E-01	-1.092E+65	-1.092E+05	-4.368E+05	1.579E+03	1.177E+07	0.000007	-1.000008
102	A	3.690E-01	5.663E-03	1.951E-01	-1.956E+10	-1.951E+00	-7.780E+00	4.994E-02	1.158E+01	0.000887	-1.212347
103	A	3.701E-01	5.297E-03	1.946E-01	-1.951E+10	-1.946E+00	-7.763E+00	2.576E-02	1.102E+01	0.002351	-1.231680
104	A	3.781E-01	6.171E-03	1.997E-01	-2.003E+10	-1.997E+00	-7.962E+00	4.538E-02	1.154E+01	0.000400	-1.216357
105	C	1.591E-04	7.518E-01	-1.461E-01	-6.056E-01	1.461E-01	3.592E+00	-3.580E-02	1.285E+05	-0.012591	0.986050
106	C	2.210E-05	2.325E-01	-2.012E-01	-2.124E+00	2.012E-02	1.011E+00	-2.392E-02	5.030E+04	-0.001981	0.974634
107	C	2.569E-05	2.365E-01	-2.183E-01	-2.146E+00	2.183E-02	1.033E+00	-1.160E-02	2.064E+04	-0.013874	0.974972
108	A	1.943E+02	2.574E+01	3.034E-01	-3.035E+65	-3.034E+05	-1.214E+06	7.967E+03	7.270E+05	0.000006	-1.000032
109	A	2.958E+02	6.888E+01	6.169E-01	-6.170E+65	-6.169E+05	-2.467E+06	3.462E+04	2.082E+06	0.000007	-1.000010
110	A	2.959E+02	6.841E+01	6.172E-01	-6.172E+65	-6.172E+05	-2.468E+06	3.161E+04	1.766E+06	0.000005	-1.000008
111	A	3.642E-01	5.613E-03	1.906E-01	-1.911E+10	-1.906E+00	-7.600E+00	6.075E-02	1.054E+01	0.000652	-1.236339
112	A	3.692E-01	5.689E-03	1.949E-01	-1.954E+10	-1.949E+00	-7.772E+00	3.650E-02	1.155E+01	0.000582	-1.213515
113	A	1.181E+02	5.164E+00	1.092E-01	-1.092E+65	-1.092E+05	-4.367E+05	1.659E+03	1.137E+07	0.000008	-1.000008
114	A	2.960E+02	6.806E+01	6.173E-01	-6.174E+65	-6.173E+05	-2.469E+06	1.964E+04	1.544E+06	0.000002	-1.000006
115	A	1.182E+02	5.190E+00	1.092E-01	-1.092E+65	-1.092E+05	-4.369E+05	1.897E+03	4.148E+06	0.000008	-1.000010
116	A	1.181E+02	5.240E+00	1.092E-01	-1.092E+65	-1.092E+05	-4.368E+05	2.939E+03	2.968E+06	0.000003	-1.000005
117	A	1.182E+02	5.280E+00	1.093E-01	-1.093E+65	-1.093E+05	-4.370E+05	1.929E+03	3.122E+07	0.000010	-1.000010
118	A	2.955E+02	6.962E+01	6.163E-01	-6.164E+65	-6.163E+05	-2.465E+06	2.888E+04	1.822E+06	0.000010	-1.000015
119	A	3.683E-01	5.657E-03	1.942E-01	-1.948E+10	-1.942E+00	-7.746E+00	3.962E-02	1.144E+01	0.000591	-1.215542
120	A	1.180E+02	5.159E+00	1.091E-01	-1.091E+65	-1.091E+05	-4.364E+05	2.507E+03	4.111E+06	0.000002	-1.000003
121	A	1.181E+02	5.242E+00	1.092E-01	-1.092E+65	-1.092E+05	-4.366E+05	1.679E+03	1.380E+08	0.000008	-1.000008
122	A	3.713E-01	5.843E-03	1.962E-01	-1.968E+10	-1.962E+00	-7.826E+00	3.926E-02	1.159E+01	0.000464	-1.212210
123	A	2.955E+02	6.987E+01	6.162E-01	-6.162E+65	-6.162E+05	-2.464E+06	4.544E+04	2.016E+06	0.000004	-1.000013
124	A	3.691E-01	6.392E-03	1.930E-01	-1.936E+10	-1.930E+00	-7.694E+00	5.376E-02	1.061E+01	0.000274	-1.237786
125	A	3.691E-01	6.393E-03	1.930E-01	-1.936E+10	-1.930E+00	-7.694E+00	5.376E-02	1.061E+01	0.000274	-1.237800
126	A	1.182E+02	5.161E+00	1.093E-01	-1.093E+65	-1.093E+05	-4.371E+05	2.493E+03	1.065E+07	0.000007	-1.000009
127	A	1.181E+02	5.220E+00	1.092E-01	-1.092E+65	-1.092E+05	-4.366E+05	2.108E+03	2.056E+07	0.000001	-1.000001
128	A	3.683E-01	5.657E-03	1.942E-01	-1.948E+10	-1.942E+00	-7.746E+00	3.962E-02	1.144E+01	0.000591	-1.215547
129	A	3.679E-01	5.642E-03	1.937E-01	-1.943E+10	-1.937E+00	-7.726E+00	4.800E-02	1.123E+01	0.000791	-1.219400
130	A	3.709E-01	5.174E-03	1.947E-01	-1.953E+10	-1.947E+00	-7.769E+00	2.775E-02	1.091E+01	0.002608	-1.234633
131	A	2.959E+02	6.856E+01	6.171E-01	-6.172E+65	-6.171E+05	-2.468E+06	2.660E+04	1.713E+06	0.000007	-1.000011
132	A	3.693E-01	5.704E-03	1.950E-01	-1.956E+10	-1.950E+00	-7.777E+00	3.451E-02	1.160E+01	0.000616	-1.212319
133	A	1.181E+02	5.183E+00	1.092E-01	-1.092E+65	-1.092E+05	-4.368E+05	2.710E+03	1.234E+06	0.000005	-1.000009
134	A	3.683E-01	5.674E-03	1.944E-01	-1.950E+10	-1.944E+00	-7.754E+00	5.086E-02	1.143E+01	0.000862	-1.215239
135	A	3.713E-01	5.843E-03	1.962E-01	-1.968E+10	-1.962E+00	-7.826E+00	3.926E-02	1.159E+01	0.000464	-1.212211
136	A	3.660E-01	5.319E-03	1.905E-01	-1.911E+10	-1.905E+00	-7.600E+00	3.386E-02	1.023E+01	0.002080	-1.252836
137	A	1.943E+02	2.574E+01	3.034E-01	-3.035E+65	-3.034E+05	-1.214E+06	7.967E+03	7.270E+05	0.000006	-1.000032
138	A	1.181E+02	5.209E+00	1.092E-01	-1.092E+65	-1.092E+05	-4.368E+05	2.262E+03	1.678E+07	0.000001	-1.000002
139	A	2.955E+02	6.987E+01	6.162E-01	-6.162E+65	-6.162E+05	-2.464E+06	4.544E+04	2.016E+06	0.000004	-1.000013
140	A	1.181E+02	5.209E+00	1.092E-01	-1.092E+65	-1.092E+05	-4.368E+05	2.262E+03	1.679E+07	0.000001	-1.000002
141	A	2.955E+02	6.962E+01	6.163E-01	-6.164E+65	-6.163E+05	-2.465E+06	2.888E+04	1.822E+06	0.000010	-1.000015
142	A	1.182E+02	5.263E+00	1.092E-01	-1.092E+65	-1.092E+05	-4.370E+05	2.082E+03	6.488E+06	0.000006	-1.000008
143	C	2.314E-05	2.352E-01	-2.045E-01	-2.148E+00	2.045E-02	1.023E+00	-1.477E-02	2.193E+04	-0.013701	0.972545
144	C	2.314E-05	2.352E-01	-2.045E-01	-2.148E+00	2.045E-02	1.023E+00	-1.477E-02	2.194E+04	-0.013710	0.972556
145	A	1.181E+02	5.191E+00	1.092E-01	-1.092E+65	-1.092E+05	-4.369E+05	2.536E+03	8.448E+06	0.000005	-1.000007
146	A	1.180E+02	5.193E+00	1.091E-01	-1.091E+65	-1.091E+05	-4.365E+05	2.705E+03	2.258E+06	0.000002	-1.000006
147	A	2.959E+02	6.856E+01	6.171E-01	-6.172E+65	-6.171E+05	-2.468E+06	2.660E+04	1.713E+06	0.000007	-1.000011
148	A	1.180E+02	5.193E+00	1.091E-01	-1.091E+65	-1.091E+05	-4.365E+05	2.705E+03	2.257E+06	0.000002	-1.000006
149	A	3.700E-01	5.816E-03	1.954E-01	-1.960E+10	-1.954E+00	-7.792E+00	3.684E-02	1.157E+01	0.000521	-1.213009
150	A	1.181E+02	5.246E+00	1.092E-01	-1.092E+65	-1.092E+05	-4.367E+05	2.728E+03	4.038E+06	0.000002	-1.000004
151	A	1.181E+02	5.174E+00	1.092E-01	-1.092E+65	-1.092E+05	-4.368E+05	1.579E+03	1.177E+07	0.000007	-1.000008
152	A	3.660E-01	5.628E-03	1.918E-01	-1.924E+10	-1.918E+00	-7.650E+00	5.383E-02	1.076E+01	0.000686	-1.230334
153	A	3.679E-01	5.642E-03	1.937E-01	-1.943E+10	-1.937E+00	-7.726E+00	4.800E-02	1.123E+01	0.000792	-1.219401
154	A	1.182E+02	5.180E+00	1.092E-01	-1.092E+65	-1.092E+05	-4.369E+05	2.648E+03	5.161E+06	0.000006	-1.000009
155	A	3.701E-01	5.297E-03	1.946E-01	-1.951E+10	-1.946E+00	-7.763E+00	2.576E-02	1.102E+01	0.002351	-1.231681
156	A	3.691E-01	5.746E-03	1.952E-01	-1.958E+10	-1.952E+00	-7.786E+00	3.668E-02	1.171E+01	0.000583	-1.210025

157	A	3.691E-01	5.746E-03	1.952E-01	-1.958E+10	-1.952E+00	-7.786E+00	3.668E-02	1.171E+01	0.000583	-1.210028
158	A	3.642E-01	5.612E-03	1.905E-01	-1.911E+10	-1.905E+00	-7.599E+00	6.076E-02	1.054E+01	0.000652	-1.236357
159	A	3.781E-01	6.171E-03	1.997E-01	-2.003E+10	-1.997E+00	-7.962E+00	4.540E-02	1.154E+01	0.000400	-1.216386
160	A	1.181E+02	5.246E+00	1.092E-01	-1.092E+65	-1.092E+05	-4.367E+05	2.728E+03	4.036E+06	0.000002	-1.000004
161	A	3.711E-01	5.881E-03	1.968E-01	-1.974E+10	-1.968E+00	-7.848E+00	4.305E-02	1.194E+01	0.000760	-1.205035
162	A	1.181E+02	5.183E+00	1.092E-01	-1.092E+65	-1.092E+05	-4.368E+05	2.710E+03	1.234E+06	0.000005	-1.000009
163	A	1.182E+02	5.190E+00	1.092E-01	-1.092E+65	-1.092E+05	-4.369E+05	1.897E+03	4.148E+06	0.000008	-1.000010
164	A	3.690E-01	5.663E-03	1.951E-01	-1.956E+10	-1.951E+00	-7.780E+00	4.994E-02	1.158E+01	0.000886	-1.212348
165	A	3.660E-01	5.628E-03	1.918E-01	-1.924E+10	-1.918E+00	-7.650E+00	5.384E-02	1.076E+01	0.000686	-1.230332
166	A	1.182E+02	5.180E+00	1.092E-01	-1.092E+65	-1.092E+05	-4.369E+05	2.648E+03	5.160E+06	0.000006	-1.000009
167	A	1.181E+02	5.164E+00	1.092E-01	-1.092E+65	-1.092E+05	-4.367E+05	1.659E+03	1.138E+07	0.000008	-1.000008
168	A	1.181E+02	5.207E+00	1.092E-01	-1.092E+65	-1.092E+05	-4.369E+05	2.330E+03	1.599E+07	0.000006	-1.000007
169	A	2.961E+02	6.799E+01	6.174E-01	-6.175E+65	-6.174E+05	-2.469E+06	2.402E+04	1.556E+06	0.000010	-1.000010
170	A	1.181E+02	5.240E+00	1.092E-01	-1.092E+65	-1.092E+05	-4.368E+05	2.940E+03	2.968E+06	0.000003	-1.000005
171	A	2.961E+02	6.799E+01	6.174E-01	-6.175E+65	-6.174E+05	-2.469E+06	2.402E+04	1.556E+06	0.000010	-1.000010
172	A	3.726E-01	5.547E-03	1.960E-01	-1.966E+10	-1.960E+00	-7.819E+00	3.905E-02	1.106E+01	0.002629	-1.229404
173	A	1.180E+02	5.159E+00	1.091E-01	-1.091E+65	-1.091E+05	-4.364E+05	2.508E+03	4.108E+06	0.000002	-1.000003
174	A	3.711E-01	5.881E-03	1.968E-01	-1.974E+10	-1.968E+00	-7.848E+00	4.305E-02	1.194E+01	0.000760	-1.205040
175	A	1.182E+02	5.208E+00	1.092E-01	-1.092E+65	-1.092E+05	-4.369E+05	2.410E+03	1.143E+07	0.000007	-1.000007
176	A	3.660E-01	5.319E-03	1.905E-01	-1.911E+10	-1.905E+00	-7.600E+00	3.386E-02	1.023E+01	0.002080	-1.252835
177	A	1.181E+02	5.191E+00	1.092E-01	-1.092E+65	-1.092E+05	-4.369E+05	2.537E+03	8.450E+06	0.000005	-1.000007
178	A	3.709E-01	5.174E-03	1.947E-01	-1.953E+10	-1.947E+00	-7.769E+00	2.775E-02	1.091E+01	0.002608	-1.234633
179	A	1.181E+02	5.186E+00	1.092E-01	-1.092E+65	-1.092E+05	-4.368E+05	2.273E+03	1.726E+07	0.000006	-1.000008
180	A	3.683E-01	5.674E-03	1.944E-01	-1.950E+10	-1.944E+00	-7.755E+00	5.085E-02	1.143E+01	0.000862	-1.215217
181	A	3.692E-01	5.689E-03	1.949E-01	-1.954E+10	-1.949E+00	-7.772E+00	3.651E-02	1.155E+01	0.000582	-1.213513
182	A	1.182E+02	5.208E+00	1.092E-01	-1.092E+65	-1.092E+05	-4.369E+05	2.411E+03	1.143E+07	0.000007	-1.000007
183	A	3.718E-01	5.463E-03	1.952E-01	-1.958E+10	-1.952E+00	-7.788E+00	4.103E-02	1.088E+01	0.002529	-1.233743
184	A	3.718E-01	5.463E-03	1.952E-01	-1.958E+10	-1.952E+00	-7.788E+00	4.103E-02	1.088E+01	0.002529	-1.233745
185	A	1.181E+02	5.220E+00	1.092E-01	-1.092E+65	-1.092E+05	-4.366E+05	2.108E+03	2.060E+07	0.000001	-1.000001
186	A	3.693E-01	5.704E-03	1.950E-01	-1.956E+10	-1.950E+00	-7.777E+00	3.452E-02	1.160E+01	0.000616	-1.212323
187	A	3.716E-01	5.269E-03	1.956E-01	-1.961E+10	-1.956E+00	-7.804E+00	3.753E-02	1.108E+01	0.002584	-1.228999
188	A	1.181E+02	5.207E+00	1.092E-01	-1.092E+65	-1.092E+05	-4.369E+05	2.330E+03	1.600E+07	0.000006	-1.000007
189	A	1.181E+02	5.186E+00	1.092E-01	-1.092E+65	-1.092E+05	-4.368E+05	2.274E+03	1.725E+07	0.000006	-1.000008
190	A	3.700E-01	5.816E-03	1.954E-01	-1.960E+10	-1.954E+00	-7.792E+00	3.684E-02	1.157E+01	0.000521	-1.213000
191	A	3.726E-01	5.547E-03	1.960E-01	-1.966E+10	-1.960E+00	-7.819E+00	3.906E-02	1.106E+01	0.002629	-1.229393
192	A	3.716E-01	5.269E-03	1.956E-01	-1.961E+10	-1.956E+00	-7.804E+00	3.754E-02	1.108E+01	0.002584	-1.229003
193	B	3.121E-01	9.902E-02	3.130E-01	-4.120E-01	-3.130E-01	-8.558E-01	4.526E-03	7.307E-01	0.201030	1.995911
194	B	3.122E-01	9.872E-02	3.129E-01	-4.117E-01	-3.129E-01	-8.569E-01	4.607E-03	7.282E-01	0.196712	1.990682
195	B	3.097E-01	9.948E-02	3.082E-01	-4.077E-01	-3.082E-01	-8.349E-01	4.659E-03	7.252E-01	0.204470	1.943666
196	R	2.016E-02	3.055E-02	-8.846E-01	-2.170E-13	8.846E-03	1.576E-01	-9.082E-04	1.674E-02	-1.182315	0.174593
197	B	2.420E-01	5.522E-02	1.911E-01	-2.463E-01	-1.911E-01	-5.434E-01	2.857E-03	4.870E-01	0.012066	1.307199
198	B	3.122E-01	9.872E-02	3.129E-01	-4.117E-01	-3.129E-01	-8.569E-01	4.606E-03	7.282E-01	0.196729	1.990700
199	B	3.117E-01	9.971E-02	3.118E-01	-4.115E-01	-3.118E-01	-8.484E-01	4.919E-03	7.244E-01	0.197892	1.961238
200	B	2.503E-01	5.831E-02	2.039E-01	-2.622E-01	-2.039E-01	-5.824E-01	3.379E-03	5.167E-01	0.031448	1.360793
201	B	3.121E-01	9.902E-02	3.130E-01	-4.120E-01	-3.130E-01	-8.558E-01	4.525E-03	7.307E-01	0.201024	1.995869
202	B	8.448E-03	5.412E-03	-1.630E-01	-3.782E-23	1.630E-03	2.817E-02	-1.418E-04	-8.014E-02	1.033922	0.152402
203	B	2.382E-01	5.524E-02	1.867E-01	-2.419E-01	-1.867E-01	-5.257E-01	3.274E-03	4.667E-01	0.012204	1.289982
204	B	3.040E-01	9.791E-02	2.989E-01	-3.969E-01	-2.989E-01	-8.041E-01	4.843E-03	7.238E-01	0.238586	1.906846
205	B	7.294E-03	5.655E-03	-7.029E-01	-4.952E-24	7.029E-04	2.543E-02	-2.823E-04	-1.944E-01	1.418409	0.149530
206	B	2.977E-01	8.740E-02	2.843E-01	-3.717E-01	-2.843E-01	-7.876E-01	3.193E-03	7.324E-01	0.154998	1.784980
207	B	3.117E-01	9.971E-02	3.118E-01	-4.115E-01	-3.118E-01	-8.484E-01	4.918E-03	7.244E-01	0.197874	1.961193
208	B	3.051E-01	9.655E-02	3.010E-01	-3.975E-01	-3.010E-01	-8.177E-01	5.043E-03	7.181E-01	0.222348	1.912867
209	R	7.448E-03	6.101E-03	-1.570E-01	-4.532E-23	1.570E-03	3.068E-02	-1.580E-04	-6.278E-02	-1.598212	0.134069
210	R	7.208E-03	5.672E-03	-9.680E-01	-4.704E-24	9.680E-04	2.656E-02	-3.164E-04	-1.584E-01	-2.461138	0.120122
211	R	2.016E-02	3.055E-02	-8.846E-01	-2.170E-13	8.846E-03	1.576E-01	-9.079E-04	1.676E-02	-1.182314	0.174579
212	B	3.168E-01	1.046E-01	3.226E-01	-4.271E-01	-3.226E-01	-8.721E-01	3.237E-03	8.000E-01	0.232108	2.075198
213	B	2.422E-01	3.678E-01	1.438E-01	-5.115E-01	-1.438E-01	8.960E-01	-1.036E-02	8.443E-01	0.022660	0.245808
214	R	3.283E-03	2.507E-03	-6.271E-01	-1.880E-24	6.271E-04	1.253E-02	-1.004E-04	-1.704E-01	-1.224314	0.151016
215	B	2.422E-01	3.678E-01	1.438E-01	-5.115E-01	-1.438E-01	8.960E-01	-1.036E-02	8.442E-01	0.022686	0.245801
216	B	3.097E-01	9.948E-02	3.082E-01	-4.077E-01	-3.082E-01	-8.349E-01	4.657E-03	7.252E-01	0.204496	1.943614
217	B	2.515E-01	5.945E-02	2.069E-01	-2.664E-01	-2.069E-01	-5.898E-01	3.635E-03	5.129E-01	0.029685	1.374281
218	B	8.230E-03	6.254E-03	-1.228E-01	-5.026E-23	1.228E-03	2.993E-02	-2.357E-04	-1.801E-01	1.001119	0.146798
219	B	6.957E-03	5.362E-03	-1.315E-01	-4.047E-23	1.315E-03	2.671E-02	-2.270E-04	-1.719E-01	0.164595	0.160361
220	B	2.879E-01	1.714E-01	3.902E-01	-5.616E-01	-3.902E-01	-8.751E-01	5.552E-03	1.179E+00	0.020272	2.285061
221	B	2.430E-01	5.575E-02	1.925E-01	-2.482E-01	-1.925E-01	-5.469E-01	3.421E-03	4.649E-01	0.013556	1.313080
222	B	3.190E-01	1.054E-01	3.278E-01	-4.332E-01	-3.278E-01	-8.893E-01	4.048E-03	8.118E-01	0.206767	2.072477
223	B	5.359E-03	4.074E-03	-1.136E-01	-2.937E-23	1.136E-03	2.084E-02	-1.919E-04	-1.716E-01	0.213327	0.150327
224	R	2.814E-03	2.220E-03	-5.857E-01	-1.634E-24	5.857E-04	1.122E-02	-9.273E-05	-1.716E-01	-2.097606	0.112182
225	B	2.515E-01	5.946E-02	2.069E-01	-2.664E-01	-2.069E-01	-5.898E-01	3.635E-03	5.129E-01	0.029672	1.374346
226	R	2.804E-03	2.212E-03	-5.846E-01	-1.627E-24	5.846E-04	1.119E-02	-9.245E-05	-1.716E-01	-2.094974	0.112087
227	B	2.430E-01	5.575E-02	1.925E-01	-2.482E-01	-1.925E-01	-5.470E-01	3.421E-03	4.649E-01	0.013555	1.313148
228	R	7.181E-03	6.314E-03	-1.202E-01	-5.112E-23	1.202E-03	3.007E-02	-2.440E-04	-1.645E-01	-1.856282	0.160401
229	R	2.024E-02	3.027E-02	-8.619E-01	-2.165E-13	8.619E-03	1.556E-01	-6.391E-04	6.469E-02	-1.215190	0.166214
230	B	3.051E-01	9.655E-02	3.010E-01	-3.975E-01	-3.010E-01	-8.177E-01	5.042E-03	7.181E-01	0.222373	1.912801
231	B	2.231E-01	3.357E-01	1.190E-01	-4.547E-01	-1.190E-01	8.667E-01	-1.084E-02	7.899E-01	0.028365	0.237980
232	B	2.384E-01	5.546E-02	1.868E-01	-2.423E-01	-1.868E-01	-5.255E-01	2.972E-03	4.675E-01	0.019804	1.291279
233	R	3.718E-03	2.430E-03	-5.098E-01	-1.921E-24	5.098E-04	1.176E-02	-9.418E-05	-1.390E-01	-2.263980	0.155093
234	B	2.384E-01	5.546E-02	1.868E-01	-2.423E-01	-1.868E-01	-5.254E-01	2.972E-03	4.675E-01	0.019803	1.291254
235	B	1.402E-01	1.877E-01	3.481E-01	-2.225E+00	-3.481E-02	6.116E-01	-8.216E-03	5.497E-01	0.038809	0.213227
236	B	5.372E-02	5.777E-02	1.188E-01	-5.896E-13	-1.188E-03	2.263E-01	-4.100E-03	2.040E-01	0.012009	0.175655
237	B	1.112E-01	1.343E-01	1.722E-01	-1.515E+00	-1.722E-02	4.683E-01	-8.268E-03	4.397E-01	0.006928	0.207285
238	B	3.040E-01	9.791E-02	2.990E-01	-3.969E-01	-2.990E-01	-8.042E-01	4.842E-03	7.239E-01	0.238583	1.906891
239	B	6.976E-03	5.378E-03	-1.317E-01	-4.061E-23	1.317E-03	2.678E-02	-2.276E-04	-1.719E-01	0.163638	0.160438
240	B	6.950E-03	5.395E-03	-1.369E-01	-4.026E-23	1.369E-03	2.706E-02	-2.604E-04	-1.659E-01	0.126081	0.160651
241	R	3.239E-03	2.639E-03	-7.570E-01	-1.882E-24	7.570E-04	1.358E-02	-1.128E-04	-1.721E-01	-1.358911	0.111778

242	B	2.975E-01	8.611E-02	2.835E-01	-3.696E-01	-2.835E-01	-7.894E-01	2.945E-03	7.363E-01	0.146615	1.765778
243	B	8.216E-03	6.241E-03	-1.227E-01	-5.014E-23	1.227E-03	2.987E-02	-2.352E-04	-1.802E-01	1.014221	0.146906
244	B	2.231E-01	3.357E-01	1.190E-01	-4.547E-01	-1.190E-01	8.668E-01	-1.084E-02	7.899E-01	0.028381	0.237973
245	C	2.023E-03	1.965E-03	-6.226E-01	-1.342E-24	6.226E-04	1.035E-02	-9.193E-05	-1.794E-01	-0.092382	0.469506
246	B	8.451E-03	5.415E-03	-1.631E-01	-3.784E-23	1.631E-03	2.818E-02	-1.418E-04	-8.012E-02	1.033833	0.152374
247	B	2.702E-01	6.782E-02	2.346E-01	-3.024E-01	-2.346E-01	-6.672E-01	3.465E-03	6.395E-01	0.080007	1.473828
248	R	3.130E-02	2.969E-02	-2.789E-01	-2.941E-14	2.789E-04	1.199E-01	-2.594E-03	-4.577E-02	-101.394303	0.174541
249	B	1.112E-01	1.343E-01	1.723E-01	-1.516E+00	-1.723E-02	4.683E-01	-8.269E-03	4.398E-01	0.006933	0.207294
250	B	2.503E-01	5.831E-02	2.039E-01	-2.622E-01	-2.039E-01	-5.824E-01	3.378E-03	5.168E-01	0.031518	1.360838
251	B	5.370E-02	5.774E-02	1.187E-01	-5.892E-13	-1.187E-03	2.262E-01	-4.097E-03	2.039E-01	0.012010	0.175644
252	C	2.022E-03	1.963E-03	-6.221E-01	-1.341E-24	6.221E-04	1.034E-02	-9.183E-05	-1.793E-01	-0.088578	0.468985
253	B	3.826E-01	4.706E-01	6.594E-01	-1.130E+09	-6.594E-01	-7.554E-01	4.511E-03	1.882E+00	0.225448	1.044211
254	B	3.037E-01	9.015E-02	2.939E-01	-3.841E-01	-2.939E-01	-8.152E-01	3.743E-03	7.346E-01	0.134819	1.765541
255	B	1.402E-01	1.877E-01	3.482E-01	-2.225E+00	-3.482E-02	6.117E-01	-8.216E-03	5.498E-01	0.038841	0.213227
256	R	3.274E-03	2.501E-03	-6.270E-01	-1.874E-24	6.270E-04	1.251E-02	-1.002E-04	-1.704E-01	-1.221303	0.148923
257	B	3.529E-02	4.108E-02	-3.998E-01	-4.068E-14	3.998E-04	1.659E-01	-1.814E-03	-4.732E-02	0.046052	0.150154
258	B	2.420E-01	5.522E-02	1.911E-01	-2.463E-01	-1.911E-01	-5.434E-01	2.856E-03	4.870E-01	0.012054	1.307228
259	R	7.451E-03	6.104E-03	-1.570E-01	-4.534E-23	1.570E-03	3.070E-02	-1.580E-04	-6.276E-02	-1.598085	0.134058
260	R	7.172E-03	6.306E-03	-1.201E-01	-5.105E-23	1.201E-03	3.003E-02	-2.437E-04	-1.646E-01	-1.856463	0.160242
261	B	2.879E-01	1.713E-01	3.901E-01	-5.614E-01	-3.901E-01	-8.751E-01	5.551E-03	1.178E+00	0.020226	2.284721
262	B	1.364E-01	1.781E-01	3.215E-01	-2.103E+00	-3.215E-02	5.840E-01	-6.984E-03	5.352E-01	0.012516	0.212659
263	B	1.240E-01	1.561E-01	2.436E-01	-1.804E+00	-2.436E-02	5.269E-01	-1.236E-02	4.866E-01	0.027145	0.211725
264	R	8.441E-03	6.650E-03	-8.801E-01	-5.770E-24	8.801E-04	3.012E-02	-4.691E-04	-1.238E-01	-1.138160	0.031672
265	R	3.245E-03	2.644E-03	-7.579E-01	-1.886E-24	7.579E-04	1.361E-02	-1.130E-04	-1.720E-01	-1.359896	0.112005
266	B	3.076E-01	9.099E-02	3.018E-01	-3.928E-01	-3.018E-01	-8.431E-01	3.465E-03	7.597E-01	0.139822	1.846327
267	B	4.137E-02	4.552E-02	2.470E-01	-4.799E-13	-2.470E-03	1.722E-01	-1.362E-03	-2.719E-02	0.080087	0.166843
268	R	2.250E-02	1.891E-02	1.230E-01	-2.014E-13	-1.230E-03	7.073E-02	-1.982E-03	-5.009E-02	-2.024117	0.211478
269	B	5.330E-03	4.049E-03	-1.132E-01	-2.918E-23	1.132E-03	2.072E-02	-1.908E-04	-1.716E-01	0.219056	0.150199
270	C	8.431E-03	6.505E-03	-8.008E-01	-5.705E-24	8.008E-04	2.922E-02	-4.610E-04	-1.229E-01	-0.899649	0.032970
271	B	2.382E-01	5.524E-02	1.867E-01	-2.419E-01	-1.867E-01	-5.257E-01	3.273E-03	4.668E-01	0.012209	1.289970
272	R	3.011E-02	2.738E-02	1.558E-01	-2.894E-13	-1.558E-03	1.033E-01	-2.774E-03	4.523E-03	-2.013802	0.214011
273	B	4.820E-02	4.828E-02	2.217E-01	-5.050E-13	-2.217E-03	1.843E-01	-4.060E-03	1.568E-01	0.049001	0.178676
274	R	7.243E-03	5.041E-03	-1.034E-01	-4.007E-23	1.034E-03	2.430E-02	-1.967E-04	-1.280E-01	-2.051233	0.206930
275	B	4.822E-02	4.831E-02	2.218E-01	-5.053E-13	-2.218E-03	1.844E-01	-4.063E-03	1.569E-01	0.048875	0.178691
276	R	3.966E-02	6.410E-02	-9.599E-01	-5.450E-13	9.599E-03	2.948E-01	-1.494E-03	1.534E-01	-1.264033	0.226152
277	B	2.977E-01	8.740E-02	2.843E-01	-3.717E-01	-2.843E-01	-7.876E-01	3.192E-03	7.324E-01	0.154999	1.784983
278	R	6.987E-03	5.690E-03	-8.487E-01	-4.841E-24	8.487E-04	2.615E-02	-3.153E-04	-1.403E-01	-1.308178	0.109670
279	R	6.983E-03	5.684E-03	-8.488E-01	-4.836E-24	8.488E-04	2.613E-02	-3.150E-04	-1.402E-01	-1.306947	0.109424
280	B	1.458E-02	1.257E-02	-2.100E-01	-1.047E-13	2.100E-03	5.869E-02	-4.616E-04	-1.414E-01	0.094904	0.178711
281	R	2.250E-02	1.891E-02	1.230E-01	-2.014E-13	-1.230E-03	7.072E-02	-1.982E-03	-5.011E-02	-2.024707	0.211489
282	R	3.011E-02	2.737E-02	1.558E-01	-2.893E-13	-1.558E-03	1.033E-01	-2.774E-03	4.511E-03	-2.013492	0.214023
283	R	2.339E-02	1.995E-02	1.096E-01	-2.105E-13	-1.096E-03	7.541E-02	-1.288E-03	-3.210E-02	-2.345529	0.205155
284	B	2.580E-01	5.734E-02	2.135E-01	-2.708E-01	-2.135E-01	-6.246E-01	3.584E-03	5.645E-01	0.100603	1.397659
285	C	1.412E-02	1.397E-02	1.104E-01	-1.408E-14	-1.104E-04	5.544E-02	-2.560E-03	-1.134E-01	-0.198257	0.646349
286	B	1.461E-02	1.260E-02	-2.102E-01	-1.050E-13	2.102E-03	5.880E-02	-4.624E-04	-1.413E-01	0.094377	0.178745
287	B	2.975E-01	8.611E-02	2.835E-01	-3.696E-01	-2.835E-01	-7.894E-01	2.945E-03	7.363E-01	0.146613	1.765781
288	R	1.013E-02	7.617E-03	-4.400E-01	-7.177E-24	4.400E-04	3.223E-02	-4.931E-04	-1.286E-01	-1.462382	0.178667
289	B	2.403E-01	3.545E-01	1.411E-01	-4.956E-01	-1.411E-01	8.533E-01	-7.875E-03	8.431E-01	0.009273	0.250100
290	B	3.531E-02	4.112E-02	-3.976E-01	-4.072E-14	3.976E-04	1.661E-01	-1.816E-03	-4.723E-02	0.045976	0.150149
291	R	1.533E-02	1.500E-02	-2.611E-01	-1.239E-13	2.611E-03	7.043E-02	-4.951E-04	-7.728E-02	-1.553899	0.160404
292	B	6.032E-02	6.847E-02	2.180E-01	-6.869E-14	-2.180E-04	2.730E-01	-3.621E-03	2.457E-01	0.017361	0.177472
293	R	7.250E-03	5.046E-03	-1.036E-01	-4.010E-23	1.036E-03	2.433E-02	-1.970E-04	-1.279E-01	-2.058750	0.206628
294	B	2.263E-01	3.437E-01	1.232E-01	-4.669E-01	-1.232E-01	8.818E-01	-1.458E-02	8.132E-01	0.021371	0.236570
295	B	3.371E-02	3.919E-02	-6.223E-01	-3.857E-14	6.223E-04	1.593E-01	-1.892E-03	-4.293E-02	0.060486	0.148639
296	B	2.055E-01	3.109E-01	9.720E-01	-4.081E+00	-9.720E-02	8.546E-01	-1.541E-02	7.520E-01	0.076889	0.231859
297	B	3.190E-01	1.054E-01	3.277E-01	-4.332E-01	-3.277E-01	-8.893E-01	4.047E-03	8.118E-01	0.206768	2.072433
298	B	3.037E-01	9.015E-02	2.939E-01	-3.841E-01	-2.939E-01	-8.152E-01	3.743E-03	7.346E-01	0.134826	1.765536
299	B	1.240E-01	1.561E-01	2.435E-01	-1.804E+00	-2.435E-02	5.269E-01	-1.236E-02	4.866E-01	0.027159	0.211720
300	R	3.736E-03	2.438E-03	-5.110E-01	-1.928E-24	5.110E-04	1.180E-02	-9.448E-05	-1.389E-01	-2.283783	0.156100
301	B	1.364E-01	1.781E-01	3.214E-01	-2.103E+00	-3.214E-02	5.840E-01	-6.983E-03	5.351E-01	0.012516	0.212657
302	R	2.023E-02	1.738E-02	9.979E-01	-1.838E-14	-9.979E-04	6.553E-02	-1.223E-03	-3.161E-02	-1.560848	0.202091
303	R	2.339E-02	1.995E-02	1.097E-01	-2.105E-13	-1.097E-03	7.542E-02	-1.288E-03	-3.206E-02	-2.344664	0.205185
304	B	2.175E-01	3.259E-01	1.120E-01	-4.379E-01	-1.120E-01	8.556E-01	-8.769E-03	7.750E-01	0.031057	0.236131
305	R	1.013E-02	7.618E-03	-4.399E-01	-7.178E-24	4.399E-04	3.223E-02	-4.932E-04	-1.286E-01	-1.462586	0.178692
306	R	1.097E-02	8.361E-03	-4.397E-01	-7.921E-24	4.397E-04	3.520E-02	-5.720E-04	-1.161E-01	-1.578775	0.170570
307	R	3.168E-01	1.046E-01	3.226E-01	-4.271E-01	-3.226E-01	-8.721E-01	3.237E-03	8.000E-01	0.232104	2.075201
308	R	6.532E-04	4.426E-04	-1.715E-01	-2.711E-34	1.715E-04	2.457E-03	-1.297E-05	-1.177E-01	-1.684196	0.124159
309	B	2.928E-02	2.759E-02	1.520E-01	-2.910E-13	-1.520E-03	1.043E-01	-1.779E-03	7.508E-02	0.279350	0.165538
310	B	5.230E-02	5.359E-02	2.323E-01	-5.591E-13	-2.323E-03	2.051E-01	-8.277E-03	1.688E-01	0.007086	0.176282
311	B	1.201E-02	9.789E-03	-5.493E-01	-9.240E-24	5.493E-04	4.136E-02	-6.492E-04	-1.250E-01	0.019458	0.108892
312	B	8.873E-02	1.001E-01	8.879E-01	-1.089E+01	-8.879E-03	3.647E-01	-1.325E-02	3.273E-01	0.016234	0.197540
313	B	6.957E-03	5.401E-03	-1.370E-01	-4.031E-23	1.370E-03	2.708E-02	-2.606E-04	-1.659E-01	0.127312	0.160736
314	R	2.024E-02	3.027E-02	-8.619E-01	-2.165E-13	8.619E-03	1.556E-01	-6.391E-04	6.470E-02	-1.215189	0.166214
315	B	3.357E-02	3.838E-02	-6.048E-01	-3.777E-14	6.048E-04	1.559E-01	-1.556E-03	-5.788E-02	0.044147	0.148699
316	B	3.826E-01	4.705E-01	6.594E-01	-1.130E+09	-6.594E-01	-7.555E-01	4.512E-03	1.882E+00	0.225449	1.044478
317	B	4.135E-02	4.549E-02	2.463E-01	-4.795E-13	-2.463E-03	1.721E-01	-1.361E-03	-2.728E-02	0.080089	0.166828
318	B	2.702E-01	6.781E-02	2.346E-01	-3.024E-01	-2.346E-01	-6.672E-01	3.465E-03	6.394E-01	0.079988	1.473789
319	R	8.587E-03	6.282E-03	-5.470E-01	-5.735E-24	5.470E-04	2.732E-02	-3.824E-04	-1.296E-01	-1.381352	0.179556
320	R	4.874E-04	3.882E-04	-1.704E-01	-2.178E-34	1.704E-04	2.235E-03	-1.365E-05	-1.476E-01	-1.172536	0.078940
321	R	2.483E-02	2.068E-02	1.048E-01	-2.173E-13	-1.048E-03	7.855E-02	-1.750E+01	-8.187E-02	-3.430487	0.204737
322	R	4.869E-04	3.878E-04	-1.702E-01	-2.176E-34	1.702E-04	2.232E-03	-1.363E-05	-1.476E-01	-1.172835	0.078931
323	R	8.588E-03	6.284E-03	-5.470E-01	-5.737E-24	5.470E-04	2.732E-02	-3.825E-04	-1.296E-01	-1.381399	0.179533
324	B	2.702E-01	6.781E-02	2.346E-01	-3.024E-01	-2.346E-01	-6.672E-01	3.465E-03	6.394E-01	0.079991	1.473762
325	B	4.137E-02	4.552E-02	2.470E-01	-4.800E-13	-2.470E-03	1.722E-01	-1.362E-03	-2.718E-02	0.080091	0.166836
326	B	3.826E-01	4.705E-01	6.594E-01	-1.130E+09	-6.594E-01	-7.555E-01	4.512E-03	1.882E+00	0.225430	1.044364

327	B	3.358E-02	3.839E-02	-6.042E-01	-3.779E-14	6.042E-04	1.560E-01	-1.557E-03	-5.785E-02	0.044138	0.148699
328	R	2.024E-02	3.027E-02	-8.619E-01	-2.165E-13	8.619E-03	1.556E-01	-6.391E-04	6.469E-02	-1.215188	0.166215
329	B	6.968E-03	5.410E-03	-1.372E-01	-4.038E-23	1.372E-03	2.713E-02	-2.610E-04	-1.658E-01	0.127278	0.160773
330	A	2.198E+00	6.816E+00	1.432E-01	-2.114E+21	-1.432E+01	-3.003E+01	3.150E-01	9.639E+00	108.802596	-149.911713
331	C	4.321E-04	3.683E-04	-1.634E-01	-2.049E-34	1.634E-04	2.127E-03	-1.274E-05	-1.373E-01	-0.497666	0.161383
332	B	8.873E-02	1.001E-01	8.879E-01	-1.089E+01	-8.879E-03	3.648E-01	-1.326E-02	3.273E-01	0.016236	0.197540
333	B	1.201E-02	9.790E-03	-5.493E-01	-9.241E-24	5.493E-04	4.136E-02	-6.493E-04	-1.250E-01	0.019271	0.108892
334	B	5.229E-02	5.358E-02	2.323E-01	-5.590E-13	-2.323E-03	2.050E-01	-8.277E-03	1.688E-01	0.007104	0.176279
335	B	2.928E-02	2.758E-02	1.520E-01	-2.910E-13	-1.520E-03	1.043E-01	-1.778E-03	7.508E-02	0.279364	0.165544
336	R	6.545E-04	4.434E-04	-1.717E-01	-2.718E-34	1.717E-04	2.460E-03	-1.299E-05	-1.177E-01	-1.683897	0.124303
337	B	3.168E-01	1.046E-01	3.226E-01	-4.272E-01	-3.226E-01	-8.721E-01	3.237E-03	8.000E-01	0.232105	2.075212
338	R	1.097E-02	8.360E-03	-4.396E-01	-7.920E-24	4.396E-04	3.520E-02	-5.720E-04	-1.160E-01	-1.578580	0.170614
339	R	1.012E-02	7.617E-03	-4.400E-01	-7.177E-24	4.400E-04	3.223E-02	-4.931E-04	-1.286E-01	-1.462441	0.178671
340	B	2.175E-01	3.259E-01	1.120E-01	-4.379E-01	-1.120E-01	8.556E-01	-8.769E-03	7.750E-01	0.031060	0.236131
341	R	2.339E-02	1.995E-02	1.097E-01	-2.105E-13	-1.097E-03	7.541E-02	-1.288E-03	-3.206E-02	-2.344711	0.205178
342	R	2.023E-02	1.738E-02	9.979E-01	-1.838E-14	-9.979E-04	6.554E-02	-1.224E-03	-3.161E-02	-1.560872	0.202092
343	B	1.364E-01	1.781E-01	3.215E-01	-2.103E+00	-3.215E-02	5.840E-01	-6.983E-03	5.351E-01	0.012522	0.212660
344	R	3.739E-03	2.440E-03	-5.111E-01	-1.929E-24	5.111E-04	1.181E-02	-9.453E-05	-1.389E-01	-2.286025	0.156207
345	B	1.240E-01	1.561E-01	2.435E-01	-1.804E+00	-2.435E-02	5.269E-01	-1.236E-02	4.866E-01	0.027158	0.211724
346	B	3.037E-01	9.015E-02	2.939E-01	-3.841E-01	-2.939E-01	-8.152E-01	3.743E-03	7.346E-01	0.134826	1.765550
347	R	4.218E-03	2.849E-03	-8.045E-01	-2.045E-24	8.045E-04	1.462E-02	-1.369E-04	-1.494E-01	-1.629948	0.089949
348	B	3.190E-01	1.054E-01	3.278E-01	-4.332E-01	-3.278E-01	-8.893E-01	4.047E-03	8.118E-01	0.206770	2.072455
349	B	2.055E-01	3.109E-01	9.721E-01	-4.081E+00	-9.721E-02	8.546E-01	-1.541E-02	7.520E-01	0.076892	0.231866
350	B	3.370E-02	3.917E-02	-6.231E-01	-3.855E-14	6.231E-04	1.592E-01	-1.891E-03	-4.298E-02	0.060530	0.148641
351	B	2.263E-01	3.436E-01	1.232E-01	-4.669E-01	-1.232E-01	8.817E-01	-1.458E-02	8.131E-01	0.021368	0.236571
352	R	7.249E-03	5.045E-03	-1.036E-01	-4.009E-23	1.036E-03	2.432E-02	-1.969E-04	-1.279E-01	-2.058122	0.206676
353	B	6.033E-02	6.848E-02	2.177E-01	-6.869E-14	-2.177E-04	2.730E-01	-3.622E-03	2.457E-01	0.017350	0.177470
354	R	1.533E-02	1.499E-02	-2.609E-01	-1.238E-13	2.609E-03	7.041E-02	-4.950E-04	-7.731E-02	-1.554199	0.160402
355	B	3.530E-02	4.110E-02	-3.991E-01	-4.070E-14	3.991E-04	1.660E-01	-1.815E-03	-4.728E-02	0.045974	0.150149
356	B	2.403E-01	3.545E-01	1.411E-01	-4.956E-01	-1.411E-01	8.533E-01	-7.875E-03	8.431E-01	0.009274	0.250101
357	R	1.013E-02	7.618E-03	-4.399E-01	-7.178E-24	4.399E-04	3.223E-02	-4.932E-04	-1.286E-01	-1.462461	0.178686
358	B	2.975E-01	8.611E-02	2.835E-01	-3.696E-01	-2.835E-01	-7.894E-01	2.945E-03	7.363E-01	0.146615	1.765800
359	B	1.461E-02	1.260E-02	-2.102E-01	-1.050E-13	2.102E-03	5.882E-02	-4.626E-04	-1.413E-01	0.094447	0.178752
360	C	1.412E-02	1.397E-02	1.103E-01	-1.408E-14	-1.103E-04	5.544E-02	-2.559E-03	-1.135E-01	-0.198268	0.646299
361	B	2.580E-01	5.734E-02	2.135E-01	-2.708E-01	-2.135E-01	-6.246E-01	3.584E-03	5.645E-01	0.100606	1.397670
362	R	2.339E-02	1.995E-02	1.096E-01	-2.104E-13	-1.096E-03	7.541E-02	-1.288E-03	-3.210E-02	-2.345576	0.205162
363	R	3.011E-02	2.738E-02	1.557E-01	-2.894E-13	-1.557E-03	1.033E-01	-2.774E-03	4.509E-03	-2.013704	0.214005
364	R	2.250E-02	1.891E-02	1.230E-01	-2.014E-13	-1.230E-03	7.072E-02	-1.981E-03	-5.010E-02	-2.024209	0.211493
365	B	1.458E-02	1.257E-02	-2.100E-01	-1.047E-13	2.100E-03	5.869E-02	-4.615E-04	-1.414E-01	0.094874	0.178704
366	R	6.986E-03	5.688E-03	-8.488E-01	-4.839E-24	8.488E-04	2.615E-02	-3.152E-04	-1.403E-01	-1.307769	0.109557
367	R	6.986E-03	5.688E-03	-8.486E-01	-4.839E-24	8.486E-04	2.615E-02	-3.152E-04	-1.403E-01	-1.308061	0.109630
368	B	2.977E-01	8.740E-02	2.843E-01	-3.717E-01	-2.843E-01	-7.875E-01	3.192E-03	7.324E-01	0.154998	1.784969
369	R	3.966E-02	6.410E-02	-9.599E-01	-5.450E-13	9.599E-03	2.948E-01	-1.494E-03	1.534E-01	-1.264034	0.226152
370	B	4.822E-02	4.831E-02	2.218E-01	-5.053E-13	-2.218E-03	1.844E-01	-4.062E-03	1.569E-01	0.048889	0.178692
371	R	7.246E-03	5.044E-03	-1.035E-01	-4.009E-23	1.035E-03	2.432E-02	-1.969E-04	-1.280E-01	-2.053179	0.206835
372	B	4.820E-02	4.829E-02	2.217E-01	-5.051E-13	-2.217E-03	1.843E-01	-4.060E-03	1.568E-01	0.048983	0.178679
373	R	3.011E-02	2.738E-02	1.558E-01	-2.894E-13	-1.558E-03	1.033E-01	-2.774E-03	4.521E-03	-2.013810	0.214015
374	R	2.382E-01	5.524E-02	1.867E-01	-2.419E-01	-1.867E-01	-5.257E-01	3.273E-03	4.668E-01	0.012210	1.289982
375	B	5.328E-03	4.048E-03	-1.131E-01	-2.916E-23	1.131E-03	2.072E-02	-1.908E-04	-1.716E-01	0.219768	0.150182
376	R	2.250E-02	1.891E-02	1.230E-01	-2.014E-13	-1.230E-03	7.072E-02	-1.981E-03	-5.011E-02	-2.024049	0.211476
377	B	4.137E-02	4.552E-02	2.470E-01	-4.799E-13	-2.470E-03	1.722E-01	-1.362E-03	-2.720E-02	0.080074	0.166840
378	B	3.076E-01	9.099E-02	3.018E-01	-3.928E-01	-3.018E-01	-8.431E-01	3.465E-03	7.597E-01	0.139821	1.846308
379	R	3.243E-03	2.642E-03	-7.576E-01	-1.885E-24	7.576E-04	1.360E-02	-1.130E-04	-1.721E-01	-1.359580	0.111949
380	B	1.240E-01	1.561E-01	2.435E-01	-1.804E+00	-2.435E-02	5.268E-01	-1.236E-02	4.866E-01	0.027147	0.211723
381	B	1.364E-01	1.782E-01	3.216E-01	-2.103E+00	-3.216E-02	5.840E-01	-6.984E-03	5.352E-01	0.012529	0.212664
382	B	2.879E-01	1.713E-01	3.901E-01	-5.614E-01	-3.901E-01	-8.750E-01	5.551E-03	1.178E+00	0.020204	2.284647
383	R	7.171E-03	6.305E-03	-1.201E-01	-5.104E-23	1.201E-03	3.003E-02	-2.437E-04	-1.646E-01	-1.855994	0.160260
384	R	7.450E-03	6.103E-03	-1.570E-01	-4.533E-23	1.570E-03	3.069E-02	-1.580E-04	-6.276E-02	-1.598211	0.134068
385	B	2.420E-01	5.522E-02	1.911E-01	-2.463E-01	-1.911E-01	-5.434E-01	2.856E-03	4.870E-01	0.012052	1.307239
386	B	3.529E-02	4.109E-02	-3.993E-01	-4.069E-14	3.993E-04	1.660E-01	-1.815E-03	-4.729E-02	0.046032	0.150153
387	R	3.274E-03	2.501E-03	-6.270E-01	-1.874E-24	6.270E-04	1.251E-02	-1.002E-04	-1.704E-01	-1.221113	0.148763
388	B	1.402E-01	1.877E-01	3.481E-01	-2.225E+00	-3.481E-02	6.116E-01	-8.214E-03	5.497E-01	0.038821	0.213224
389	B	3.037E-01	9.015E-02	2.939E-01	-3.841E-01	-2.939E-01	-8.152E-01	3.743E-03	7.346E-01	0.134819	1.765540
390	B	3.826E-01	4.706E-01	6.594E-01	-1.130E+09	-6.594E-01	-7.553E-01	4.511E-03	1.882E+00	0.225441	1.044200
391	C	2.022E-03	1.964E-03	-6.223E-01	-1.341E-24	6.223E-04	1.034E-02	-9.186E-05	-1.793E-01	-0.089166	0.469133
392	B	5.369E-02	5.771E-02	1.187E-01	-5.890E-13	-1.187E-03	2.261E-01	-4.095E-03	2.038E-01	0.012026	0.175642
393	B	2.503E-01	5.831E-02	2.039E-01	-2.622E-01	-2.039E-01	-5.824E-01	3.378E-03	5.167E-01	0.031523	1.360831
394	B	1.113E-01	1.343E-01	1.724E-01	-1.516E+00	-1.724E-02	4.684E-01	-8.270E-03	4.398E-01	0.006921	0.207296
395	R	3.130E-02	2.968E-02	-2.775E-01	-2.941E-14	2.775E-04	1.198E-01	-2.593E-03	-4.577E-02	-98.256345	0.174567
396	B	2.702E-01	6.782E-02	2.346E-01	-3.024E-01	-2.346E-01	-6.672E-01	3.465E-03	6.394E-01	0.080007	1.473824
397	B	8.449E-03	5.414E-03	-1.630E-01	-3.784E-23	1.630E-03	2.818E-02	-1.418E-04	-8.012E-02	1.031297	0.152334
398	C	2.023E-03	1.965E-03	-6.226E-01	-1.342E-24	6.226E-04	1.035E-02	-9.193E-05	-1.794E-01	-0.094304	0.469312
399	B	2.231E-01	3.357E-01	1.190E-01	-4.547E-01	-1.190E-01	8.668E-01	-1.084E-02	7.899E-01	0.028376	0.237978
400	B	8.216E-03	6.241E-03	-1.227E-01	-5.014E-23	1.227E-03	2.987E-02	-2.352E-04	-1.802E-01	1.014111	0.146916
401	B	2.975E-01	8.611E-02	2.835E-01	-3.696E-01	-2.835E-01	-7.894E-01	2.945E-03	7.363E-01	0.146614	1.765781
402	R	3.241E-03	2.640E-03	-7.571E-01	-1.883E-24	7.571E-04	1.359E-02	-1.129E-04	-1.721E-01	-1.359248	0.111879
403	B	6.955E-03	5.399E-03	-1.370E-01	-4.029E-23	1.370E-03	2.707E-02	-2.606E-04	-1.659E-01	0.126095	0.160675
404	B	6.967E-03	5.370E-03	-1.316E-01	-4.054E-23	1.316E-03	2.674E-02	-2.273E-04	-1.719E-01	0.163954	0.160403
405	B	3.040E-01	9.792E-02	2.990E-01	-3.969E-01	-2.990E-01	-8.042E-01	4.842E-03	7.239E-01	0.238591	1.906998
406	B	1.112E-01	1.343E-01	1.722E-01	-1.515E+00	-1.722E-02	4.682E-01	-8.266E-03	4.397E-01	0.006929	0.207289
407	B	5.371E-02	5.774E-02	1.188E-01	-5.893E-13	-1.188E-03	2.262E-01	-4.097E-03	2.039E-01	0.012019	0.175651
408	B	1.402E-01	1.877E-01	3.481E-01	-2.225E+00	-3.481E-02	6.116E-01	-8.214E-03	5.497E-01	0.038804	0.213222
409	B	2.384E-01	5.546E-02	1.868E-01	-2.423E-01	-1.868E-01	-5.254E-01	2.971E-03	4.675E-01	0.019801	1.291231
410	R	3.720E-03	2.431E-03	-5.098E-01	-1.921E-24	5.098E-04	1.176E-02	-9.420E-05	-1.390E-01	-2.266355	0.155256
411	B	2.384E-01	5.546E-02	1.868E-01	-2.423E-01	-1.868E-01	-5.255E-01	2.972E-03	4.675E-01	0.019804	1.291300

412	B	2.231E-01	3.357E-01	1.190E-01	-4.547E-01	-1.190E-01	8.667E-01	-1.084E-02	7.899E-01	0.028369	0.237982
413	B	3.051E-01	9.655E-02	3.010E-01	-3.975E-01	-3.010E-01	-8.177E-01	5.042E-03	7.181E-01	0.222388	1.912844
414	R	2.024E-02	3.027E-02	-8.619E-01	-2.165E-13	8.619E-03	1.556E-01	-6.391E-04	6.469E-02	-1.215188	0.166216
415	R	7.178E-03	6.312E-03	-1.202E-01	-5.110E-23	1.202E-03	3.005E-02	-2.439E-04	-1.645E-01	-1.855835	0.160378
416	B	2.430E-01	5.575E-02	1.925E-01	-2.482E-01	-1.925E-01	-5.470E-01	3.421E-03	4.649E-01	0.013557	1.313157
417	R	2.806E-03	2.213E-03	-5.849E-01	-1.629E-24	5.849E-04	1.119E-02	-9.251E-05	-1.716E-01	-2.097988	0.112134
418	B	2.515E-01	5.945E-02	2.069E-01	-2.664E-01	-2.069E-01	-5.898E-01	3.635E-03	5.129E-01	0.029652	1.374310
419	R	2.815E-03	2.221E-03	-5.858E-01	-1.635E-24	5.858E-04	1.123E-02	-9.276E-05	-1.716E-01	-2.094948	0.112165
420	B	5.350E-03	4.066E-03	-1.135E-01	-2.931E-23	1.135E-03	2.080E-02	-1.916E-04	-1.716E-01	0.214568	0.150276
421	B	3.190E-01	1.054E-01	3.278E-01	-4.332E-01	-3.278E-01	-8.893E-01	4.048E-03	8.118E-01	0.206767	2.072470
422	B	2.430E-01	5.575E-02	1.925E-01	-2.482E-01	-1.925E-01	-5.469E-01	3.421E-03	4.649E-01	0.013550	1.313103
423	B	2.879E-01	1.714E-01	3.902E-01	-5.616E-01	-3.902E-01	-8.751E-01	5.552E-03	1.179E+00	0.020280	2.285049
424	B	6.960E-03	5.365E-03	-1.315E-01	-4.050E-23	1.315E-03	2.672E-02	-2.271E-04	-1.719E-01	0.164520	0.160379
425	B	8.228E-03	6.251E-03	-1.228E-01	-5.023E-23	1.228E-03	2.992E-02	-2.356E-04	-1.801E-01	1.005669	0.146857
426	B	2.515E-01	5.945E-02	2.069E-01	-2.664E-01	-2.069E-01	-5.898E-01	3.635E-03	5.129E-01	0.029697	1.374280
427	B	3.097E-01	9.948E-02	3.082E-01	-4.077E-01	-3.082E-01	-8.349E-01	4.657E-03	7.252E-01	0.204492	1.943576
428	B	2.422E-01	3.678E-01	1.438E-01	-5.115E-01	-1.438E-01	8.960E-01	-1.036E-02	8.443E-01	0.022686	0.245800
429	R	3.280E-03	2.505E-03	-6.269E-01	-1.878E-24	6.269E-04	1.253E-02	-1.003E-04	-1.704E-01	-1.223861	0.150625
430	B	2.422E-01	3.678E-01	1.438E-01	-5.115E-01	-1.438E-01	8.960E-01	-1.036E-02	8.443E-01	0.022667	0.245806
431	B	3.168E-01	1.046E-01	3.226E-01	-4.272E-01	-3.226E-01	-8.721E-01	3.237E-03	8.000E-01	0.232108	2.075210
432	R	2.016E-02	3.055E-02	-8.846E-01	-2.170E-13	8.846E-03	1.576E-01	-9.079E-04	1.676E-02	-1.182308	0.174585
433	R	7.449E-03	6.102E-03	-1.570E-01	-4.532E-23	1.570E-03	3.068E-02	-1.580E-04	-6.278E-02	-1.598099	0.134060
434	B	3.051E-01	9.655E-02	3.010E-01	-3.975E-01	-3.010E-01	-8.177E-01	5.043E-03	7.181E-01	0.222345	1.912862
435	B	3.117E-01	9.971E-02	3.118E-01	-4.115E-01	-3.118E-01	-8.485E-01	4.918E-03	7.244E-01	0.197878	1.961243
436	B	2.977E-01	8.740E-02	2.843E-01	-3.717E-01	-2.843E-01	-7.876E-01	3.193E-03	7.324E-01	0.154998	1.784978
437	B	7.293E-03	5.654E-03	-7.028E-01	-4.951E-24	7.028E-04	2.543E-02	-2.822E-04	-1.944E-01	1.417580	0.149529
438	B	3.040E-01	9.791E-02	2.989E-01	-3.968E-01	-2.989E-01	-8.041E-01	4.843E-03	7.238E-01	0.238585	1.906755
439	B	2.382E-01	5.524E-02	1.867E-01	-2.419E-01	-1.867E-01	-5.257E-01	3.274E-03	4.668E-01	0.012204	1.289984
440	B	8.448E-03	5.412E-03	-1.630E-01	-3.783E-23	1.630E-03	2.817E-02	-1.418E-04	-8.014E-02	1.035086	0.152413
441	B	3.121E-01	9.902E-02	3.130E-01	-4.120E-01	-3.130E-01	-8.558E-01	4.525E-03	7.307E-01	0.201027	1.995940
442	B	2.503E-01	5.831E-02	2.039E-01	-2.622E-01	-2.039E-01	-5.824E-01	3.379E-03	5.167E-01	0.031458	1.360800
443	B	3.117E-01	9.971E-02	3.118E-01	-4.115E-01	-3.118E-01	-8.484E-01	4.919E-03	7.244E-01	0.197884	1.961222
444	B	3.122E-01	9.872E-02	3.129E-01	-4.117E-01	-3.129E-01	-8.569E-01	4.606E-03	7.282E-01	0.196727	1.990657
445	B	2.420E-01	5.522E-02	1.911E-01	-2.463E-01	-1.911E-01	-5.434E-01	2.856E-03	4.870E-01	0.012064	1.307203
446	R	2.016E-02	3.055E-02	-8.846E-01	-2.170E-13	8.846E-03	1.576E-01	-9.081E-04	1.674E-02	-1.182312	0.174592
447	B	3.097E-01	9.948E-02	3.082E-01	-4.077E-01	-3.082E-01	-8.349E-01	4.659E-03	7.252E-01	0.204477	1.943696
448	B	3.122E-01	9.872E-02	3.130E-01	-4.117E-01	-3.130E-01	-8.569E-01	4.607E-03	7.282E-01	0.196715	1.990723
449	B	3.121E-01	9.902E-02	3.130E-01	-4.120E-01	-3.130E-01	-8.558E-01	4.526E-03	7.307E-01	0.201026	1.995882
450	C	3.124E-03	2.530E-03	-6.789E-01	-1.851E-24	6.789E-04	1.284E-02	-1.041E-04	-1.580E-01	-0.733828	0.193119
451	B	2.690E-01	3.206E-02	2.242E-01	-2.563E-01	-2.242E-01	-7.687E-01	5.214E-03	6.711E-01	0.008443	1.096039
452	A	9.483E-01	1.189E+00	3.742E-01	-4.932E+10	-3.742E+00	-1.021E+01	1.435E-01	6.488E+00	21.411879	-30.497729
453	R	4.212E-03	2.844E-03	-8.036E-01	-2.040E-24	8.036E-04	1.459E-02	-1.367E-04	-1.493E-01	-1.623430	0.087169
454	C	3.124E-03	2.530E-03	-6.794E-01	-1.851E-24	6.794E-04	1.284E-02	-1.041E-04	-1.582E-01	-0.738058	0.191396
455	B	2.700E-01	3.105E-02	2.246E-01	-2.557E-01	-2.246E-01	-7.743E-01	5.856E-03	6.771E-01	0.009385	1.087998
456	B	2.190E+00	6.869E+00	1.425E-01	-2.112E+21	-1.425E+01	-2.954E+01	2.934E-01	9.645E+00	119.469338	152.734503
457	C	6.790E-03	5.216E-03	-8.924E-01	-4.324E-24	8.924E-04	2.443E-02	-3.222E-04	-1.272E-01	-0.670456	0.193991
458	C	6.793E-03	5.218E-03	-8.924E-01	-4.326E-24	8.924E-04	2.444E-02	-3.224E-04	-1.272E-01	-0.669447	0.194657
459	A	9.618E-01	1.215E+00	3.807E-01	-5.022E+10	-3.807E+00	-1.037E+01	1.198E-01	6.393E+00	16.511309	-22.484465
460	R	4.216E-03	2.847E-03	-8.039E-01	-2.043E-24	8.039E-04	1.460E-02	-1.368E-04	-1.494E-01	-1.631973	0.089792
461	R	8.437E-03	6.643E-03	-8.790E-01	-5.764E-24	8.790E-04	3.009E-02	-4.687E-04	-1.237E-01	-1.133156	0.030660
462	C	8.427E-03	6.504E-03	-8.026E-01	-5.702E-24	8.026E-04	2.923E-02	-4.610E-04	-1.229E-01	-0.902281	0.031848
463	A	2.182E+00	6.769E+00	1.419E-01	-2.096E+21	-1.419E+01	-2.967E+01	2.974E-01	9.630E+00	117.668835	-208.199190
464	C	4.317E-04	3.679E-04	-1.632E-01	-2.047E-34	1.632E-04	2.124E-03	-1.272E-05	-1.373E-01	-0.497493	0.161270
465	B	1.253E-03	7.057E-04	-1.863E-01	-5.194E-34	1.863E-04	3.568E-03	-1.864E-05	-1.251E-01	0.305456	0.160295
466	B	2.190E+00	6.869E+00	1.425E-01	-2.112E+21	-1.425E+01	-2.954E+01	2.933E-01	9.645E+00	119.437398	152.659663
467	B	2.190E+00	6.869E+00	1.425E-01	-2.112E+21	-1.425E+01	-2.954E+01	2.934E-01	9.645E+00	119.370202	152.850810
468	C	8.428E-03	6.505E-03	-8.019E-01	-5.703E-24	8.019E-04	2.923E-02	-4.610E-04	-1.229E-01	-0.901434	0.032215
469	R	8.438E-03	6.645E-03	-8.793E-01	-5.766E-24	8.793E-04	3.010E-02	-4.688E-04	-1.238E-01	-1.134790	0.030995
470	A	9.618E-01	1.215E+00	3.807E-01	-5.022E+10	-3.807E+00	-1.037E+01	1.198E-01	6.393E+00	16.511251	-22.483293
471	C	6.792E-03	5.217E-03	-8.923E-01	-4.325E-24	8.923E-04	2.444E-02	-3.223E-04	-1.272E-01	-0.669589	0.194571
472	C	6.792E-03	5.218E-03	-8.924E-01	-4.325E-24	8.924E-04	2.444E-02	-3.223E-04	-1.272E-01	-0.669843	0.194327
473	B	2.190E+00	6.869E+00	1.425E-01	-2.112E+21	-1.425E+01	-2.954E+01	2.934E-01	9.645E+00	119.460873	152.740249
474	B	2.700E-01	3.105E-02	2.246E-01	-2.557E-01	-2.246E-01	-7.743E-01	5.856E-03	6.771E-01	0.009385	1.087997
475	C	3.124E-03	2.531E-03	-6.794E-01	-1.851E-24	6.794E-04	1.284E-02	-1.042E-04	-1.582E-01	-0.738181	0.191432
476	C	8.430E-03	6.505E-03	-8.014E-01	-5.703E-24	8.014E-04	2.922E-02	-4.610E-04	-1.229E-01	-0.900608	0.032556
477	R	8.440E-03	6.647E-03	-8.796E-01	-5.767E-24	8.796E-04	3.011E-02	-4.689E-04	-1.238E-01	-1.136302	0.031293
478	R	4.211E-03	2.844E-03	-8.036E-01	-2.040E-24	8.036E-04	1.459E-02	-1.367E-04	-1.493E-01	-1.623960	0.087673
479	C	3.123E-03	2.529E-03	-6.787E-01	-1.850E-24	6.787E-04	1.283E-02	-1.041E-04	-1.581E-01	-0.734410	0.192884
480	R	7.207E-03	5.671E-03	-9.680E-01	-4.703E-24	9.680E-04	2.656E-02	-3.163E-04	-1.584E-01	-2.460264	0.120105
481	B	2.640E-01	3.453E-02	2.207E-01	-2.553E-01	-2.207E-01	-7.449E-01	3.397E-03	7.012E-01	0.006532	1.118153
482	B	2.714E-01	3.035E-02	2.283E-01	-2.586E-01	-2.283E-01	-7.917E-01	2.696E-03	7.836E-01	0.013917	1.112036
483	B	2.640E-01	3.453E-02	2.207E-01	-2.553E-01	-2.207E-01	-7.449E-01	3.396E-03	7.012E-01	0.006532	1.118145
484	A	2.205E+00	6.794E+00	1.439E-01	-2.118E+21	-1.439E+01	-3.037E+01	2.831E-01	9.643E+00	59.100787	-169.785555
485	B	2.714E-01	3.035E-02	2.283E-01	-2.586E-01	-2.283E-01	-7.917E-01	2.696E-03	7.836E-01	0.013915	1.112033
486	A	2.205E+00	6.794E+00	1.439E-01	-2.118E+21	-1.439E+01	-3.037E+01	2.831E-01	9.643E+00	59.059295	-169.712517
487	B	2.714E-01	3.035E-02	2.283E-01	-2.586E-01	-2.283E-01	-7.917E-01	2.696E-03	7.836E-01	0.013915	1.112034
488	A	2.184E+00	6.799E+00	1.420E-01	-2.100E+21	-1.420E+01	-2.961E+01	3.100E-01	9.647E+00	176.463595	-504.505520
489	B	2.640E-01	3.453E-02	2.207E-01	-2.553E-01	-2.207E-01	-7.448E-01	3.396E-03	7.012E-01	0.006533	1.118145
490	R	8.589E-01	1.343E+00	3.279E-01	-4.623E+10	-3.279E+00	-7.744E+00	1.012E-01	6.536E+00	-27.659012	24.969069
491	B	2.714E-01	3.035E-02	2.283E-01	-2.586E-01	-2.283E-01	-7.917E-01	2.696E-03	7.836E-01	0.013916	1.112036
492	B	2.640E-01	3.453E-02	2.207E-01	-2.553E-01	-2.207E-01	-7.449E-01	3.396E-03	7.012E-01	0.006532	1.118152

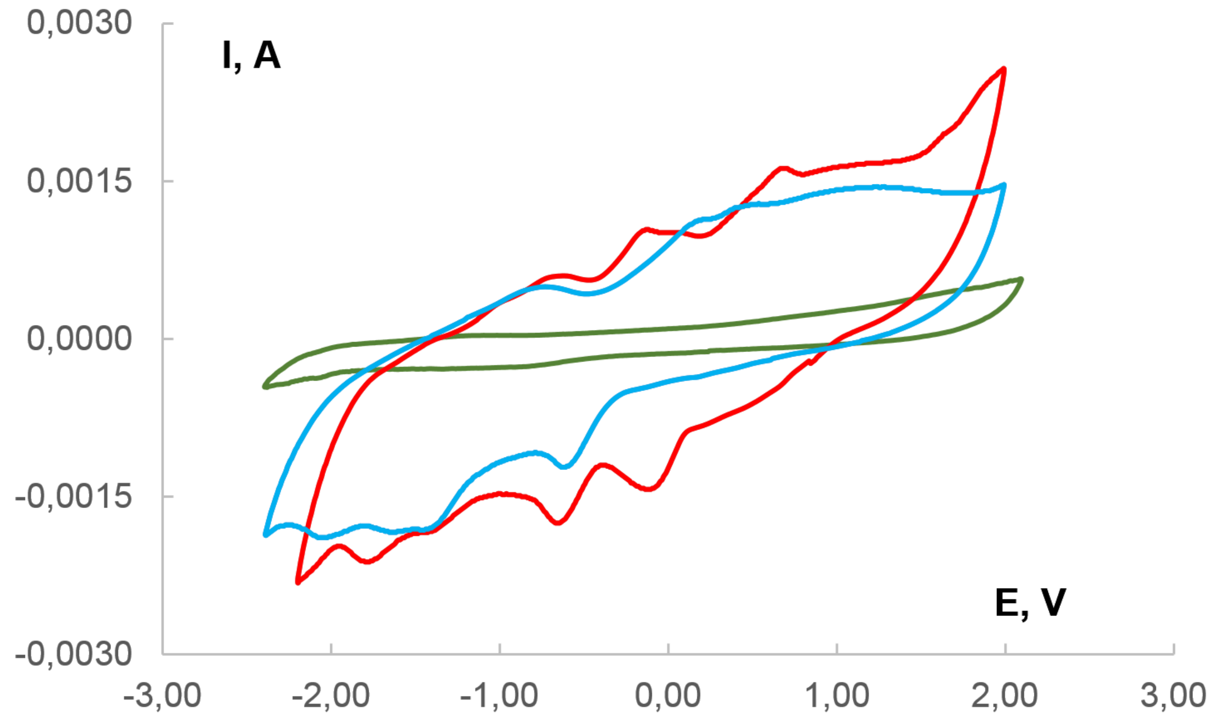

Figure S2. Comparison of CVs of paste electrodes in 0.10 M Bu_4NPF_6 in acetonitrile at a scan rate of 20 mV/s: red curve - complex in paste, blue curve - precursor, green curve - background (blank).

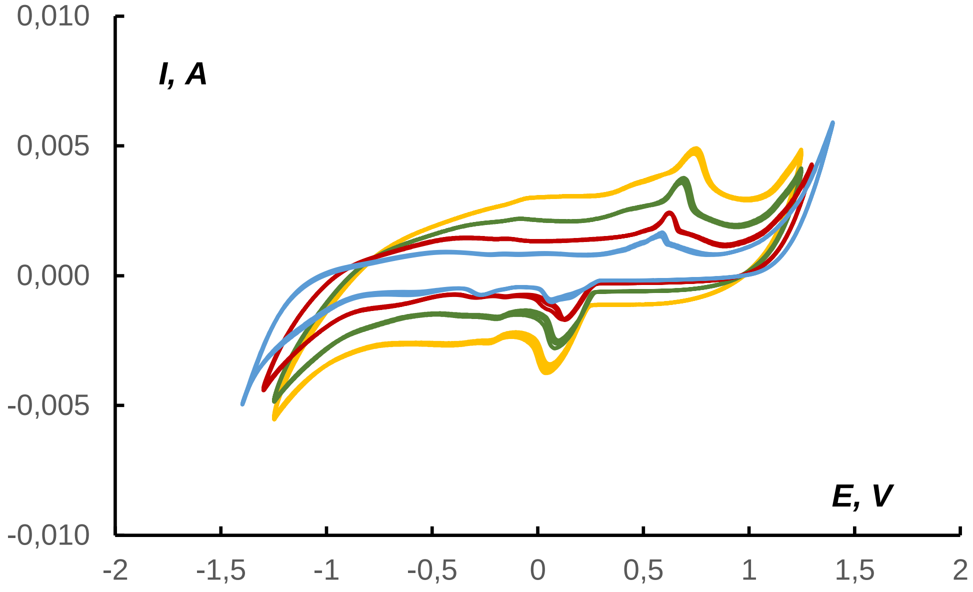

Figure S3. Reproducibility of CV curves (2-4 cycles) for **1** in the paste electrode in aqueous solution of 1.0 M $KHCO_3$ at different scan rates (mV/s): blue line – 10, red – 20, green – 50, yellow – 100.

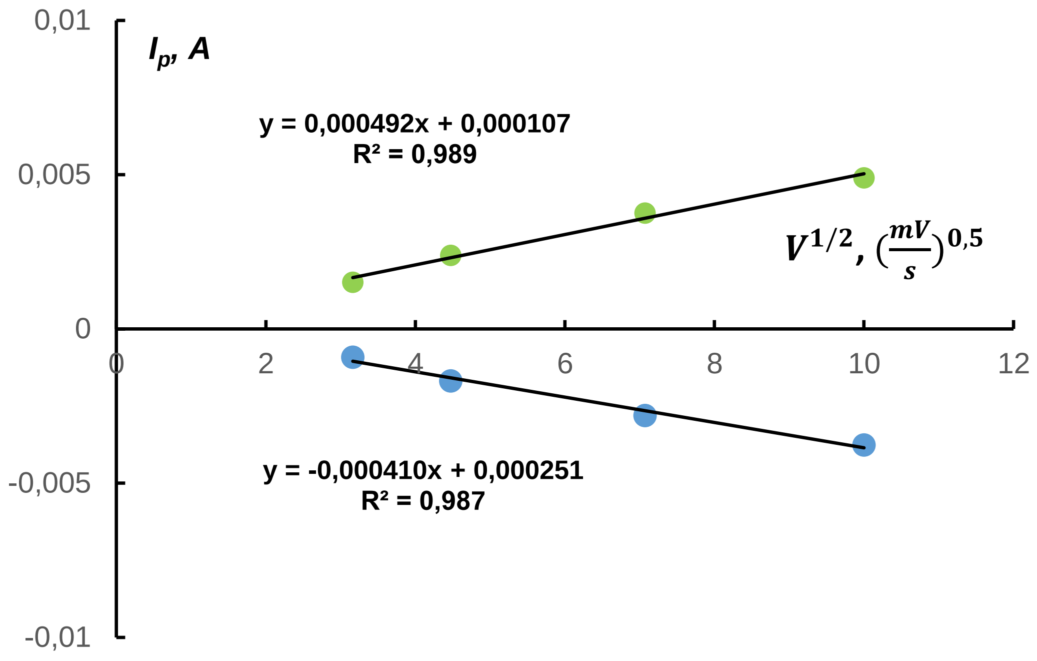

Figure S4. The dependences of peak currents on the square root of scan rate for anodic (green circles) and cathodic (blue circles) peak currents. The primary data presented in Fig. S3.

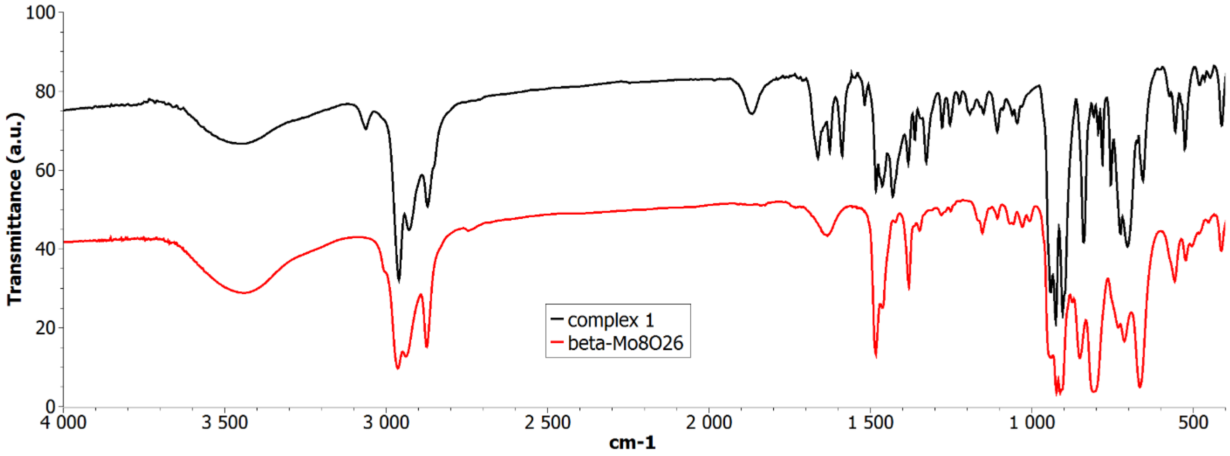

Figure S5. IR spectra of 1 (black curve) and $(Bu_4N)_4[\beta\text{-}Mo_8O_{26}]$ (red curve).

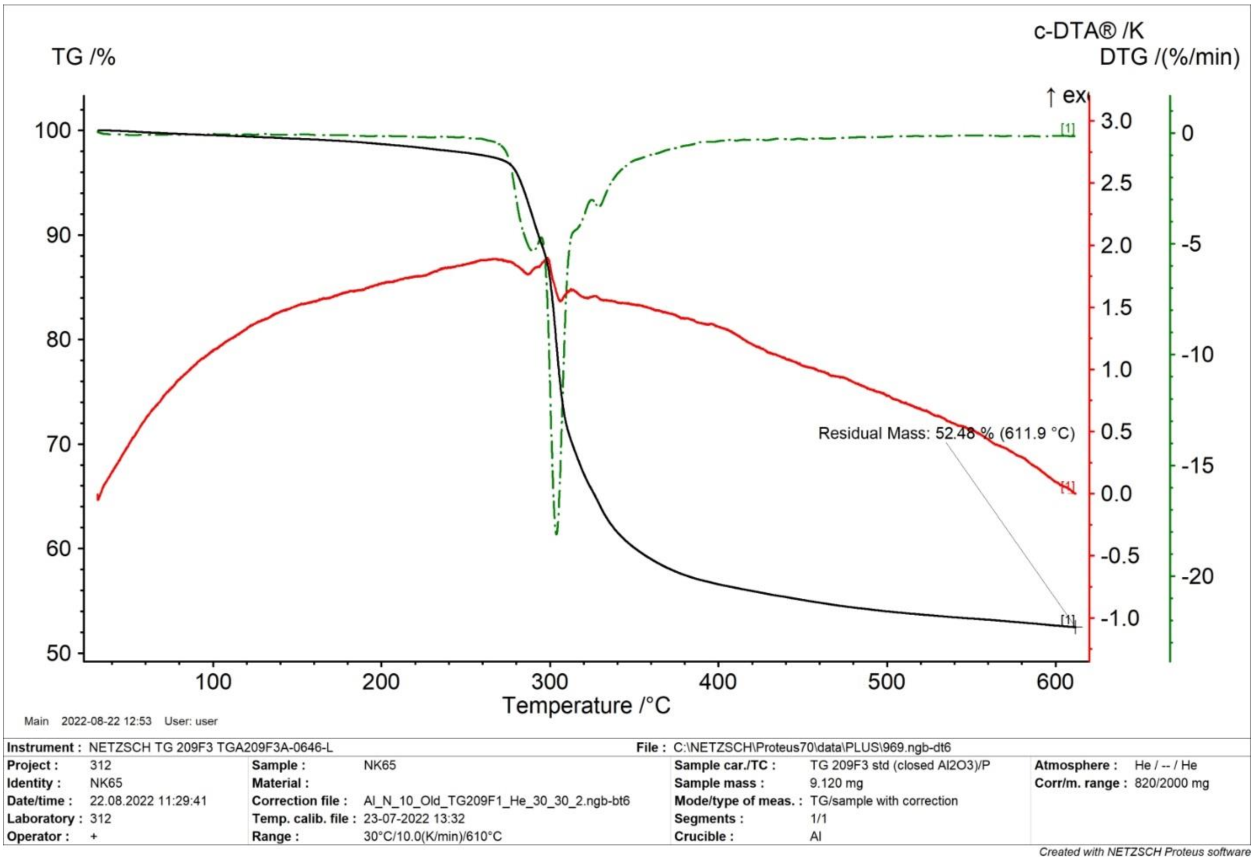

Figure S6. TGA data for **1**.
